# Evidence for a Role of the Lateral Ectoderm in *Drosophila* Mesoderm Invagination

**DOI:** 10.3389/fcell.2022.867438

**Published:** 2022-04-25

**Authors:** Hanqing Guo, Shicheng Huang, Bing He

**Affiliations:** Department of Biological Sciences, Dartmouth College, Hanover, NH, United States

**Keywords:** epithelial folding, ventral furrow formation, gastrulation, apical constriction, compression, buckling

## Abstract

The folding of two-dimensional epithelial sheets into specific three-dimensional structures is a fundamental tissue construction mechanism in animal development. A common mechanism that mediates epithelial folding is apical constriction, the active shrinking of cell apices driven by actomyosin contractions. It remains unclear whether cells outside of the constriction domain also contribute to folding. During *Drosophila* mesoderm invagination, ventrally localized mesoderm epithelium undergoes apical constriction and subsequently folds into a furrow. While the critical role of apical constriction in ventral furrow formation has been well demonstrated, it remains unclear whether, and if so, how the laterally localized ectodermal tissue adjacent to the mesoderm contributes to furrow invagination. In this study, we combine experimental and computational approaches to test the potential function of the ectoderm in mesoderm invagination. Through laser-mediated, targeted disruption of cell formation prior to gastrulation, we found that the presence of intact lateral ectoderm is important for the effective transition between apical constriction and furrow invagination in the mesoderm. In addition, using a laser-ablation approach widely used for probing tissue tension, we found that the lateral ectodermal tissues exhibit signatures of tissue compression when ablation was performed shortly before the onset of mesoderm invagination. These observations led to the hypothesis that in-plane compression from the surrounding ectoderm facilitates mesoderm invagination by triggering buckling of the mesoderm epithelium. In support of this notion, we show that the dynamics of tissue flow during mesoderm invagination displays characteristic of elastic buckling, and this tissue dynamics can be recapitulated by combining local apical constriction and global compression in a simulated elastic monolayer. We propose that *Drosophila* mesoderm invagination is achieved through epithelial buckling jointly mediated by apical constriction in the mesoderm and compression from the neighboring ectoderm.

## Introduction

Epithelial folding is a common morphogenetic mechanism that mediates the conversion of 2-dimensional cell sheets into more convoluted, 3-dimensional structures. Epithelial folding is often mediated by local constriction of the apical side of the cell sheet, which is driven by contractions of apically localized actin and non-muscle myosin II (“myosin”) networks and results in bending of the sheet towards the basal direction (Sawyer et al., 2010). While the mechanisms that activate apical constriction have been well elucidated, the way how “in-plane” constriction forces generated at the apical surface drives “out-of-the-plane” bending of the tissue is not fully understood.

**FIGURE 1:**
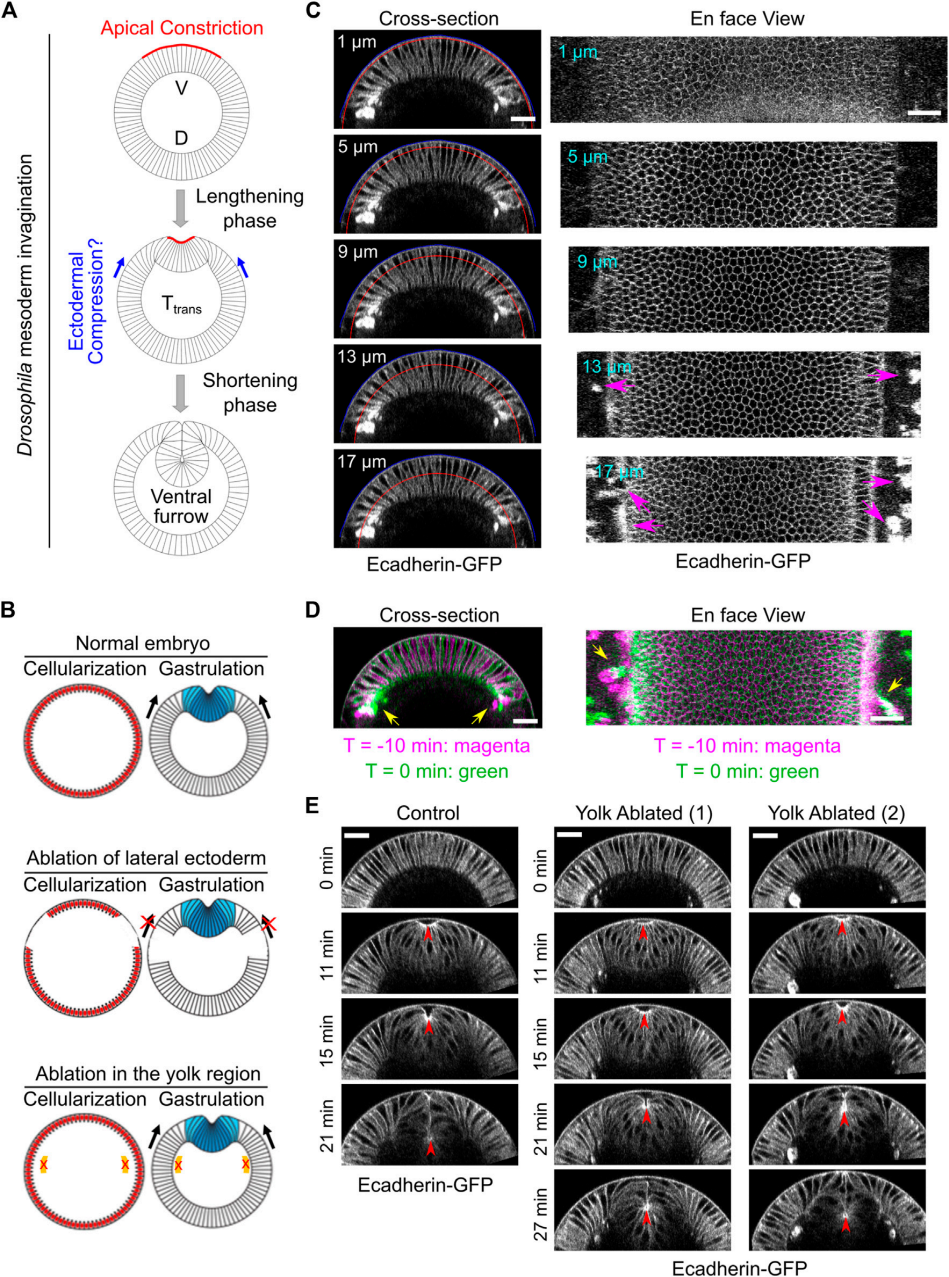
Laser disruption of cell formation in the lateral ectodermal region prior to gastrulation. **(A)** Schematics illustrating mesoderm invagination during *Drosophila* gastrulation (given the important role of apical constriction in the process of ventral furrow formation, in all images shown in this paper, the apical side of the mesoderm epithelium is facing up). While the role of apical constriction in mesoderm invagination has been well documented, it remains unclear whether the surrounding ectodermal tissue also contributes to mesoderm invagination. It has been postulated that the ectoderm may facilitate mesoderm invagination by exerting compressive stresses on the mesoderm epithelium. V: ventral; D: dorsal. **(B)** Schematics illustrating the strategy to test the requirement for the surrounding ectoderm in mesoderm invagination. In order to disrupt cell formation in the lateral ectoderm, a focused 920 nm fs laser beam was used to disrupt cleavage furrows in the lateral regions of the embryo during cellularization. Similar laser treatment in the yolk region of the embryos was performed as a control. Laser ablation was performed during cellularization and the treated embryo was imaged during gastrulation. **(C)** Cross-section and en face views showing the basal location of the “burn marks” after laser disruption in the lateral ectodermal region. No burn marks are observed at the level of the vitelline membrane. Blue and red curves in the cross-section views indicate the vitelline membrane and the position where the projections were made for the en face views, respectively. Magenta arrows: burn marks. **(D)** Overlay of the signal at T = −10 and 0 min (onset of gastrulation) showing the movement of burn marks before gastrulation (yellow arrows). **(E)** An example control embryo without laser treatment and two examples showing ventral furrow formation after laser treatment in the yolk region. Only a mild delay in furrow invagination was observed. Red arrowheads indicate the apex of the ventral most cells. All scale bars: 25 μm.

*Drosophila* ventral furrow formation during gastrulation provides an excellent model for studying epithelial folding (reviewed in (Gilmour et al., 2017; Gheisari et al., 2020; Martin, 2020)). During the first 20 min of gastrulation, the presumptive mesoderm epithelium localized at the ventral side of the embryo (18-cell wide along the DV axis and ∼60-cell long along the AP axis) undergo apical constriction and folds into a furrow along the A-P axis (Leptin and Grunewald, 1990; Sweeton et al., 1991). Ventral furrow formation proceeds in two phases (Sweeton et al., 1991). During the first ∼10 min, the ventral cells undergo apical constriction and elongate apical-basally (the “lengthening phase”). At the end of the lengthening phase, a shallow apical indentation is formed at the constricting site, but the cell apex still remains near the surface of the embryo. In the next ∼10 min, cells remain apically constricted and rapidly internalize as they shorten back to a wedge-like morphology (“the shortening phase”) (Figure 1A).

The molecular mechanism that leads to apical constriction has been well characterized (reviewed in (Martin, 2020)). In response to the dorsal-ventral patterning information, two ventrally expressed zygotic transcription factors, Twist and Snail, activate the apical recruitment of RhoGEF2 through a G-protein coupled receptor pathway (Parks and Wieschaus, 1991; Costa et al., 1994; Kolsch et al., 2007; Manning et al., 2013; Kerridge et al., 2016). RhoGEF2 in turn activates myosin at the apical surface by activating Rho1 and its effector, Rho-associated kinase (Rok) (Barrett et al., 1997; Hacker and Perrimon, 1998; Nikolaidou and Barrett, 2004; Dawes-Hoang et al., 2005; Martin et al., 2009; Mason et al., 2013; Vasquez et al., 2014). Activated myosin assembles into bipolar mini-filaments that bind to F-actin and form a contractile actomyosin network at the cell apex. The apical actomyosin network undergoes stochastic, pulsed contractions, which powers the constriction of the cell apices by pulling on apical adherens junctions (AJs) (Martin et al., 2009; Mason et al., 2013).

The essential role of apical constriction in ventral furrow formation has been well documented. Genetic or pharmacological disruption of actomyosin activities inhibits apical constriction and results in failure in ventral furrow formation (reviewed in (Gheisari et al., 2020; Martin, 2020)). However, accumulating evidence suggests that apical constriction does not directly drive furrow invagination. It has long been noted that apical constriction and rapid invagination occurs in partially distinct phases, suggesting that apical constriction cannot fully account for furrow invagination (Sweeton et al., 1991; Krajcovic and Minden, 2012; Polyakov et al., 2014; Rauzi et al., 2015). Along this vein, several additional mechanisms have been recently elucidated. First, it has been shown that myosin accumulated at the lateral membranes of constricting cells (“lateral myosin”) facilitates furrow invagination, presumably by exerting tension along the apical-basal axis of the cell (Gracia et al., 2019; John and Rauzi, 2021). Second, successful invagination of ventral furrow requires inactivation of myosin enriched at the basal side of the ventral cells to facilitate “relaxation” of the basal cortices (Polyakov et al., 2014; Krueger et al., 2018). Finally, the ectodermal tissues surrounding the mesoderm can also influence mesoderm invagination. Recent work from Rauzi et al. found that blocking the movement of the lateral ectoderm by anchoring ectodermal cell apices to the vitelline membrane blocks ventral furrow invagination (Rauzi et al., 2015). A similar disruption of ventral furrow formation has also been observed when actomyosin contractility in the lateral ectoderm was ectopically upregulated (Perez-Mockus et al., 2017). These results demonstrate that an increase in ectodermal resistance can inhibit mesoderm invagination.

In a recent study, we show that the requirement for actomyosin contractility during ventral furrow formation is stage-dependent (Guo et al., 2022). Acute loss of actomyosin contractility during most of the lengthening phase results in immediate relaxation of the constricted tissue, but similar treatment near or after a transition phase (T_trans_) close to the lengthening-shortening transition does not impede invagination. This binary response to myosin inhibition indicates that the mesoderm epithelium is mechanically bistable during gastrulation. In addition, it suggests that additional mechanical inputs other than apical constriction are involved in the actual invagination step. Interestingly, a number of modeling studies predict that mesoderm invagination requires additional mechanical input from outside of the mesoderm, such as “pushing” forces from the surrounding ectodermal tissue (Muñoz et al., 2007; Conte et al., 2009; Allena et al., 2010; Brodland et al., 2010) (Figure 1A). However, direct evidence demonstrating the mechanical contribution of the ectoderm to mesoderm invagination has not been previously demonstrated.

Here, we developed experimental approaches to test the requirement of the ectoderm in mesoderm invagination. Through laser-mediated disruption of designated cell groups, we show that the presence of the ectodermal cells flanking the mesoderm is important for an effective transition from apical constriction to invagination. In addition, using laser ablation, we show that the lateral ectoderm presents feature of tissue compression shortly before the transition phase. These observations raise the possibility that ectodermal compression may facilitate mesoderm invagination by promoting buckling of the mesoderm epithelium. Consistent with this notion, we observed a steep acceleration of tissue flow immediately after the transitional phase, which is a hallmark of elastic buckling. Using computer modeling, we further show that a combined action of local apical constriction and global compression can recapitulate the observed buckling-like tissue dynamics in a simulated elastic monolayer. Together, our combined experimental and computational analyses reveal an intimate coupling between the tissue autonomous and nonautonomous mechanisms during a tissue folding process. We propose that during *Drosophila* mesoderm invagination, ectodermal compression functions in concert with the active cell shape change in the mesoderm to trigger buckling of the mesoderm epithelium.

## Results

### Development of a Laser-Mediated Approach to Disrupt Lateral Ectodermal Cells Before Gastrulation

In order to test the potential function of the ectoderm in mesoderm invagination, we first asked whether mesoderm invagination requires the presence of adjacent ectoderm. To address this question, we sought to develop an approach to specifically disrupt the ectodermal cells that surround the mesoderm before the onset of gastrulation. We took advantage of the special form of cleavage (“cellularization”) in early *Drosophila* embryos, which occurs immediately before ventral furrow formation. During cellularization, plasma membrane furrows invaginate from the embryo surface and partition the peripherally localized syncytial nuclei into individual cells (Mazumdar and Mazumdar, 2002). We used a 920 nm fs laser with a high laser power to disrupt the invaginating cleavage furrows in the lateral ectodermal regions during cellularization (Figure 1B; Methods). By the time when cellularization is normally completed, the prospective mesoderm formed properly, but the surrounding lateral ectodermal cells were severely disrupted (Figure 1C). The interruption of cell formation is likely due to tissue cauterization, as indicated by the appearance of bright, autofluorescence “burn marks” in the laser treated regions as has been reported in previous literatures (Rauzi et al., 2015; de Medeiros et al., 2020).

The formation of burn marks raised the concern whether tissue cauterization would cause anchoring of the ectodermal tissue to the vitelline membrane. If so, this approach would generate a non-motile boundary for the mesoderm and block mesoderm invagination, as previously demonstrated (Rauzi et al., 2015). This would be the case regardless of whether ectoderm actively contribute to mesoderm invagination or it is merely passively dragged by the invaginating mesoderm. Several lines of evidence indicate that our approach did not cause anchoring of the tissue to the vitelline membrane. The major difference between the approach we used and the one used by Rauzi et al., 2015 is the location of the tissue where the laser treatment was imposed. Rauzi et al. targeted the laser to the apical side of the tissue, adjacent to the vitelline membrane. The resulting cauterization of the tissue caused anchoring of the tissue to the vitelline membrane, presumably by fusion of the tissue with the vitelline membrane. In our approach, we targeted the laser to the basal region of the invaginating cleavage furrows instead of the apical side of the tissue. As a result, the “burn marks” were not located at the apical side of the cells, and there was no sign of tissue fusion with the vitelline membrane (Figure 1C). This is further confirmed by the observation that the burn marks could move before the onset of gastrulation, which indicates that the tissue is not anchored to the vitelline membrane (Figure 1D).

Another concern about the laser cauterization approach is whether the heat generated by the laser treatment has a global, non-specific effect on embryo development. To test this, we performed a control experiment where we treated the yolk region of the embryo with the identical approach (Figure 1B). Despite the appearance of burn marks in the treated yolk region, cellularization and mesoderm invagination proceeded normally, with only a mild reduction in the rate of ventral furrow formation (Figure 1E). These results suggest that heat generated by the laser treatment does not have a prominent adverse impact on the developmental process.

### Laser Disruption of the Lateral Ectoderm Results in Reduced Rate of Apical Constriction and a Pause of Mesoderm Invagination at the Transitional State

**FIGURE 2:**
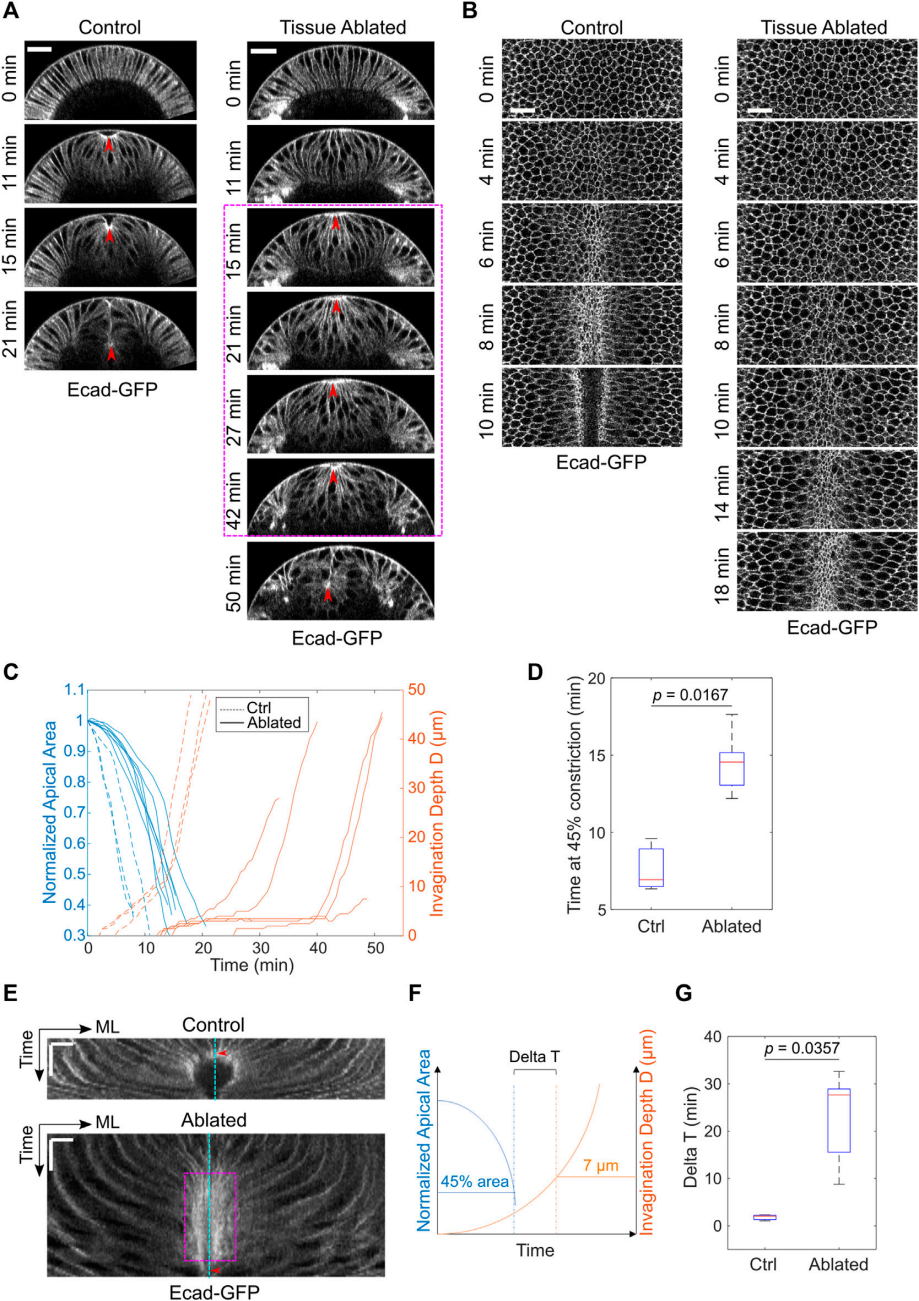
Laser disruption of the lateral ectoderm results in a pause of mesoderm invagination at T_trans_. **(A)** Cross-section views showing the delayed ventral furrow invagination in ablated embryos. Red arrowheads indicate the apex of the ventral most cells. Magenta box highlights the pause near T_trans_. Scale bars: 25 μm. **(B)** Surface projection views showing apical constriction in control and ectoderm-disrupted embryos. Scale bars: 20 μm. **(C)** Invagination depth D and the apical area of the constricting cells in control (*N* = 3) and ablated (*N* = 7) embryos during the course of ventral furrow formation. **(D)** Time for the constricting cells to achieve 45% constriction after the onset of gastrulation in control and ectoderm-disrupted embryos. **(E)** Kymographs of apical cell membrane on the ventral surface illustrating the pause near T_trans_ in ectoderm-disrupted embryos (magenta box). ML, medial-lateral. Cyan line: ventral midline. Red arrowheads indicate the time when the constricting cells disappear from the surface view. Scale bars: horizontal, 10 μm; vertical: 10 min. **(F)** Diagram illustrating how Delay at T_trans_ (Delta T) is defined. Delta T is defined as the time difference between the time when cell reaches 45% constriction and the time when invagination depth D reaches 7 μm. **(G)** Comparison of Delta T between control (*N* = 3) and ectoderm-disrupted embryos (*N* = 5). Statistical comparisons were performed using two-sided Wilcoxon rank sum test.

Next, we examined the impact of laser disruption of the lateral ectoderm on mesoderm invagination. The mesodermal cells still underwent apical constriction despite the severe disruption of the lateral ectodermal tissues (Figures 2A,B). The rate of apical constriction, however, was reduced by two-fold compared to the control, non-treated embryos (Figures 2B–D). The cause of the effect on apical constriction rate is unknown. In addition to the reduced rate of apical constriction, we observed a striking delay at the transition stage before the tissue invaginated (Figures 2A,C). In two out of seven laser-treated cases, the mesodermal tissue did not invaginate before the onset of cell division, which disrupted apical constriction altogether (Grosshans and Wieschaus, 2000; Seher and Leptin, 2000). The prolonged arrest at the transition stage can be readily appreciated by generating a kymograph of tissue movement on the ventral surface of the embryo (Figure 2E). In the control embryos, the constricting cells were promptly internalized and disappeared from the surface view when their apical area reduced to approximately 40–50% of its original size (Figure 2E, red arrowhead). In the ablated embryo, however, the constricting cells remained at the surface for a much longer time after they achieved similar level of apical constriction (Figure 2E, magenta box). To further quantify this phenotype, we measured the interval between the time when the constricting domain had reached 45% constriction and the time when the embryo reached an invagination depth D of 7 μm (“Delta T,” Figure 2F). This approach allowed us to focus on the delay at the transition phase instead of the difference in the rate of apical constriction. Delta T increased from 4.7 ± 1.5 min in the control embryos to 22.9 ± 9.5 min in the ablated embryos (mean ± standard deviation, control: *N* = 3 embryos, ablated: *N* = 5 embryos, Figure 2G). Note that Delta T was underestimated for the ablated embryos because those that failed to reach a D of 7 μm (two out of seven embryos) were not included in the calculation. Together, these results demonstrate that the presence of intact lateral ectodermal cells are important for a smooth transition from apical constriction to invagination during ventral furrow formation.

### Reducing the Rate of Apical Constriction *Per Se* Does Not Cause a Pause at the Transitional State

**FIGURE 3:**
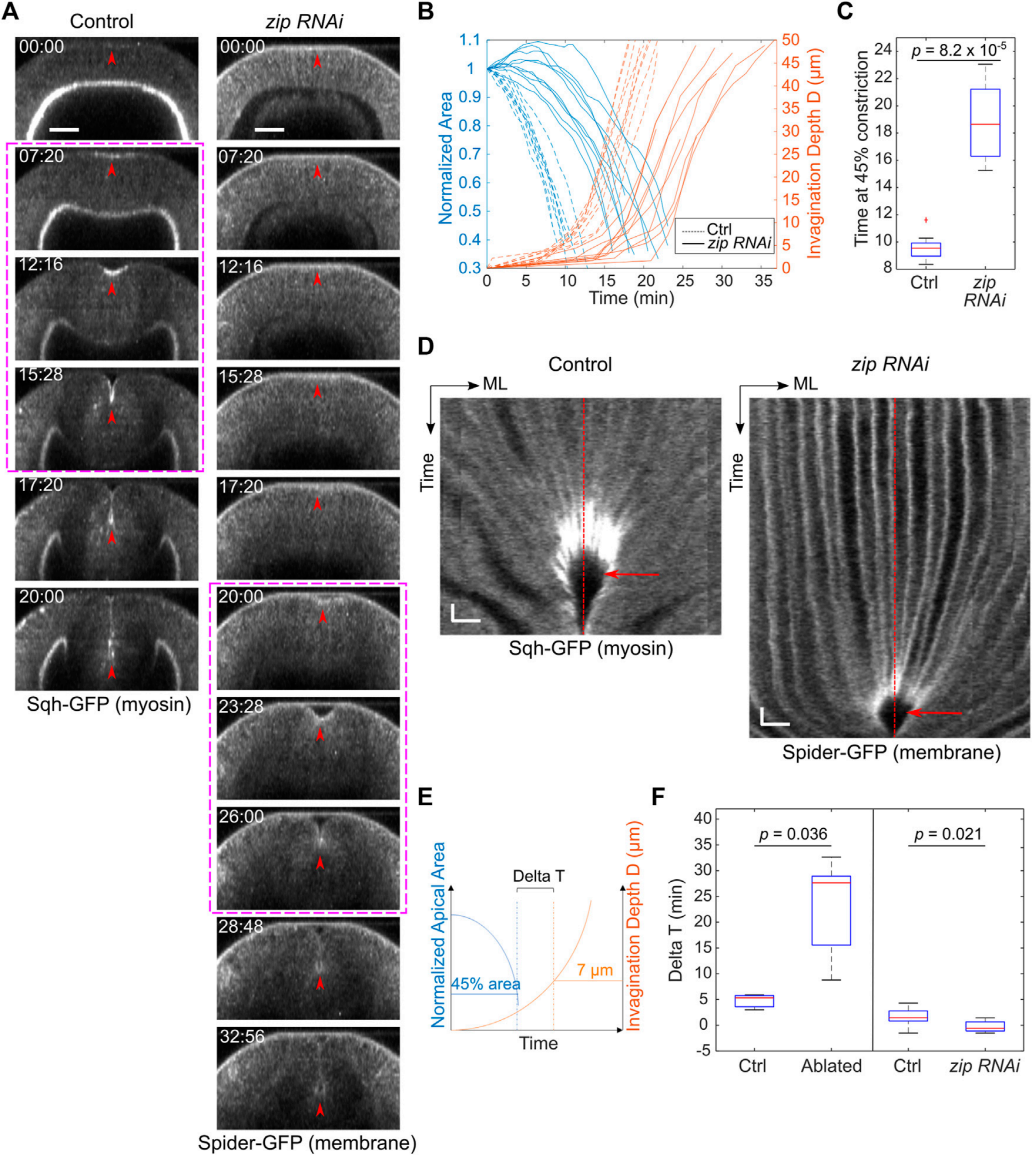
*zip RNAi* embryos show reduced rate of apical constriction but not delay at T_trans_. **(A)** Cross-sections showing ventral furrow formation in a *zip RNAi* embryo and a control embryo. *zip RNAi* embryos show a much-reduced rate of apical constriction, but the subsequent transition into rapid invagination after T_trans_ is not affected (magenta boxes). Red arrowheads indicate the apex of the ventral most cells. Scale bars: 25 μm. **(B)** Invagination depth D (orange lines) and cell apical area (blue lines) in *zip RNAi* (solid) and control (dotted) embryos. Time zero represents the onset of gastrulation, which is defined by the time point when obvious apical cell shape change happens at the ventral side of the embryo. Note that in some *zip RNAi* embryos, apical domain of the ventral cells first undergoes a transient expansion before constriction starts. **(C)** Time when the constriction domain in *zip RNAi* and control embryos reaches 45% constriction. Student’s t-test; *zip RNAi*: *N* = 8 embryos; control: *N* = 9 embryos. **(D)** Kymograph showing no obvious delay at the transitional stage (red arrow) in *zip RNAi* embryos despite the reduced rate of apical constriction. ML, medial-lateral. Red dashed lines: ventral midline. Scale bars: horizontal, 10 μm; vertical: 80 s **(E**,**F)** Comparison of Delta T between the control (*N* = 9) and *zip RNAi* (*N* = 8) embryos. *zip RNAi* embryos showed no increase in Delta T despite the reduced rate of apical constriction similar to that in embryos subjected to laser disruption of lateral ectodermal cells. Delta T in control and laser-treated embryos from Figure 2 is replotted here for comparison. Statistical comparisons were performed using two-sided Wilcoxon rank sum test.

The laser disruption of lateral ectoderm may directly impact the transition between apical constriction and invagination. Alternatively, the pause at T_trans_ may be a secondary consequence of the two-fold reduction of the rate of apical constriction. To test the second possibility, we examined whether reducing myosin heavy chain Zipper (Zip) in early embryos by maternal knockdown, which has been previously shown to reduce the rate of apical constriction (He et al., 2014), would result in a similar pause at T_trans_. In *zip RNAi* embryos, despite the ∼ 2-fold reduction in the rate of apical constriction compared with the control embryos, there was no delay at the transition between apical constriction and invagination, and the subsequent invagination appeared normal (Figure 3). The lack of defects at T_trans_ in *zip RNAi* embryos is in sharp contrast to the pronounced delay at T_trans_ when lateral ectodermal cells were disrupted. Thus, the delay in T_trans_ in ectoderm-disrupted embryos is not caused by the reduced rate of apical constriction. Together, these observations suggest that mechanical contributions from the surrounding ectoderm are important for promoting the mesoderm to transition from apical constriction to invagination.

### Using Laser Ablation to Detect Compressive Stresses in Embryonic Epithelia

**FIGURE 4:**
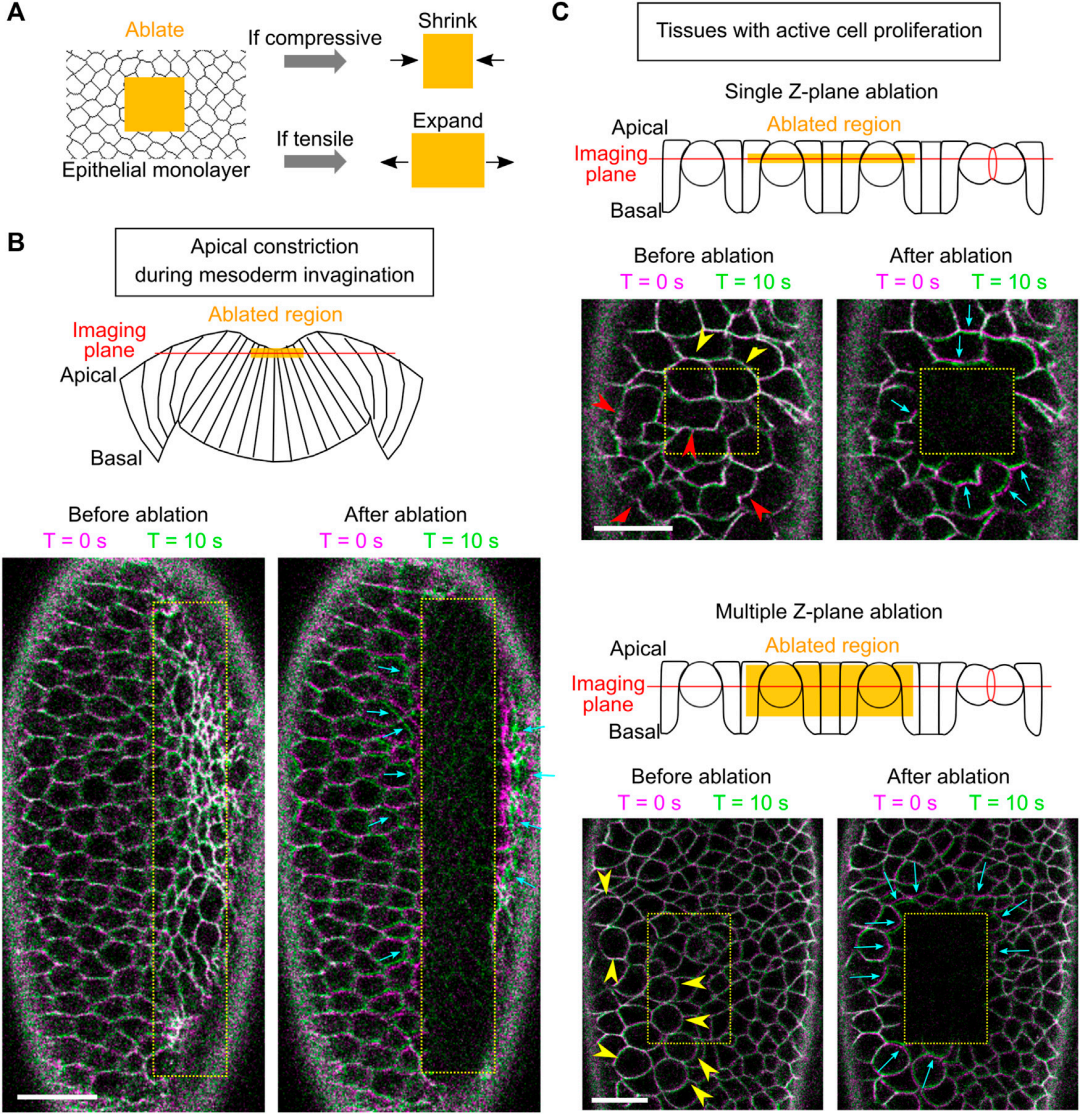
Using laser ablation to detect tissue compression in developing *Drosophila* embryos. **(A)** Predicted outcome of laser ablation when the tissue is tensile or compressive. **(B)** Laser ablation in the mesodermal tissue during apical constriction. Ablation within the constriction domain results in recoil of the surrounding cells away from the ablated region (cyan arrows). The movement of tissues is indicated by overlaying membrane signals (Ecadherin-GFP) at T = 0 s and at T = 10 s (same in panel **C**). T = 0 in the “After ablation” panels mark the time immediately after ablation. **(C)** Laser ablation within a region of the ectoderm in a stage 8–9 embryos where multiple cells are undergoing cell division. Cell division is indicated by mitotic rounding (yellow arrowheads) or the appearance of cleavage furrows (red arrowheads). Top: laser ablation is performed at a single z-plane approximately at the middle of the cell height. Bottom: laser ablation is performed at a series of z positions from 4 to 22 μm deep from the surface with a step size of 2 μm. In both cases, the surrounding cells moved towards the ablated region (cyan arrows) immediately after laser ablation. All scale bars: 20 μm.

The way how the ectoderm may contribute to mesoderm invagination remains unclear. Our recent work demonstrates that the lateral ectoderm undergoes apicobasal shrinkage during gastrulation. Interestingly, this process still occurs when mesoderm invagination is inhibited, suggesting that ectodermal shortening is not merely a consequence of mesoderm invagination and may thereby serve as an independent mechanical input that facilitates mesoderm invagination (Guo et al., 2022). In theory, apical-basal shortening of the ectodermal cells will result in tissue compression in the planar direction if the cell volume remains constant. If this is the case, ectodermal compression may function together with apical constriction to facilitate mesoderm invagination by providing pushing forces on the mesoderm epithelium. To test these hypotheses, we first sought to determine whether ectodermal compression is present during ventral furrow formation. While multiple techniques have been developed to measure mechanical properties in cells, including the compression modulus (Taubenberger et al., 2020), methods for detecting tissue compression in live embryos have not been well established. Laser ablation has been widely used to detect and measure tensile stresses in cells and tissues (Hutson et al., 2003). It has been previously shown that when laser intensity is properly controlled, ablation using a femtosecond near-infrared laser can physically disrupt certain subcellular structures, such as cortical actomyosin networks, without rapturing the plasma membrane (Rauzi et al., 2008; Rauzi and Lenne, 2015). When the ablated region is under tension, an outward recoil of the surrounding cells will be observed. The recoil velocity is determined by the magnitude of the tension and viscosity of the media (cytoplasm) around the structures (Hutson et al., 2003). We reasoned that if a similar approach is used to treat tissues under compression, disruption of mechanical integrity of the cells in the laser-treated region should cause the region to shrink, thereby providing a readout for the presence of compressive stresses (Figure 4A).

First, we sought to validate the approach by carrying out laser ablation in tissues that are known to be under tension. To this end, we targeted the mesoderm primordium during apical constriction by scanning a 920 nm fs laser with a relatively high laser intensity across a region of interest within the constriction domain (Figure 4B; Methods). As expected, after the laser treatment, the treated region expanded immediately as the surrounding cells underwent outward recoil (Figure 4B, cyan arrows) (Martin et al., 2010; Jodoin et al., 2015). The laser intensity was carefully tuned such that it was not too strong to cause obvious damage on the cell membrane, but strong enough to cause tissue recoil. The lack of damage on the plasma membrane is indicated by the lack of burn marks on the tissue or any sign of plasma membrane repair (Figure 4B) and the readily recovery of the E-cadherin signal on the plasma membrane (Supplementary Figure S1). These observations confirm that our laser ablation approach can detect tissue tension without generating obvious damage on the cell membranes.

Next, we performed laser ablation on tissues that were expected to be under compression. During cell division, cells round up from their original columnar shape. The pressure generated by the cells undergoing mitotic rounding and cytokinesis is expected to increase compressive stresses in the epithelium (Taubenberger et al., 2020). We therefore tested the effect of laser ablation in Stage 8–9 embryos at regions where cells are undergoing active mitosis (Figure 4C, arrowheads) (Foe, 1989). In the first approach, we performed laser ablation at a single z-plane at approximately the middle of the cell height (Figure 4C, top). In the second approach, we ablated the tissue at multiple z-planes, covering a depth of 4–22 μm from the surface of the tissue (Figure 4C, bottom). No signs of plasma membrane damage were observed in either approach. In both cases, we observed immediate shrinkage of the laser-treated region as the surrounding cells underwent inward recoil (Figure 4C, cyan arrows; Supplementary Movie S1). This tissue response is in contrast to the tensile response observed in the apical constriction domain. Instead, it is consistent with the expected outcome for tissues under compression. Together, these experiments suggest that laser ablation can be used to detect compressive stresses in developing embryos.

### Laser Ablation Reveals Signs of Tissue Compression in the Lateral Ectoderm Prior to Mesoderm Invagination

**FIGURE 5:**
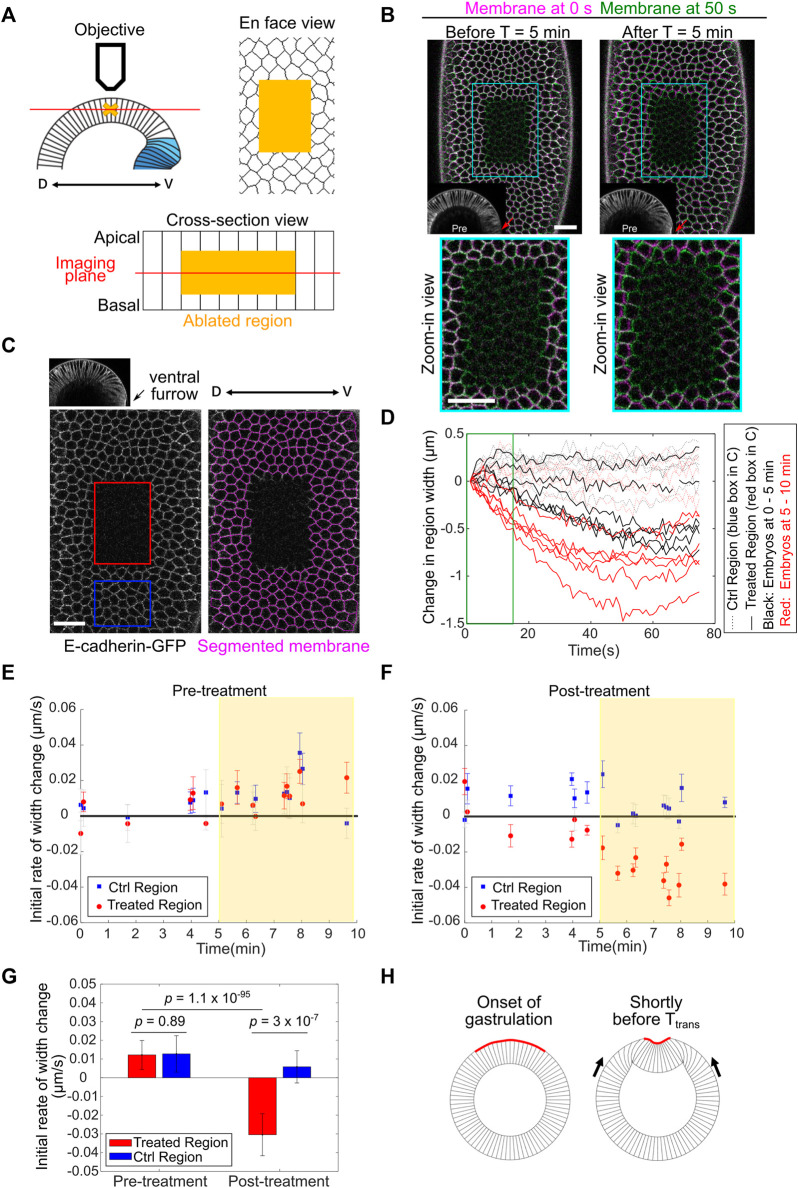
Detection of in-plane compressive stresses in the lateral ectoderm before T_trans_. **(A)** Cartoon depicting the experimental setup for laser ablation to detect compressive stresses. The middle region of the ectodermal cells (yellow cross and yellow shades) was ablated using a 920 nm fs laser. Post-ablation tissue movement was recorded by single-plane (red line) fast imaging. **(B)** Examples showing the immediate response of the tissue after laser ablation carried out during 0–5 min (left) or 5–10 min (right) of gastrulation (ventral furrow formation). Cell membrane signals immediately after ablation (0 s, magenta) or 50 s (green) after ablation are overlayed. Red arrows: ventral midline. Scale bar: 20 μm. **(C)** Left: single plane image acquired immediately after laser cutting. Red: laser-treated region. Blue: control region. Right: tracked cell membrane. Scale bar: 20 μm. **(D)** Width change of the control and laser-treated regions over time in embryos ablated during 0–5 min (*n* = 6 embryos) or 5–10 min (*n* = 6 embryos) of gastrulation. The first 15 s of the curves (green box) were used to calculate the initial rate of width change as the readout of tissue response. **(E**,**F)** Initial rate of width change before **(E)** and after **(F)** laser treatment. Error bar is 95% confidence interval of the linear fitting of the initial recoil curve. An immediate shrinkage of the cut region is evident in embryos at the stage 5–10 min into gastrulation (yellow shaded region). **(G)** Average initial rate of width change in embryos between 5–10 min into gastrulation. Error bar shows standard deviation (s.d.); *n* = 10 embryos. Student’s t-test. **(H)** Cartoon illustrating the buildup of ectodermal compression after the onset of gastrulation but before T_trans_.

Using the laser ablation approach, we then asked whether we could detect compressive stresses in the ectodermal tissue during gastrulation. To this end, we specifically targeted an ectodermal region on the lateral side of the embryo, covering approximately 5 × 9 cells and encompassing the depth of 9–27 μm from the apical side of the cell (Figure 5A; Methods). The laser treatment of the entire volume took approximately 3 s and covered the middle half of the cell height. Following laser ablation, time lapse movies at a single focal plane ∼17 μm deep from the apical surface were acquired to determine the tissue response (Figure 5A; Methods). We focused our analysis at the stage of late cellularization and early gastrulation, before the beginning of rapid ectodermal tissue movement caused by ventral furrow invagination (∼10 min after the onset of gastrulation). We found that the tissue response to laser ablation was sensitive to the stage of the embryo. Laser ablation during the first 5 min of gastrulation did not result in obvious tissue shrinkage over a 1-min time course. In contrast, evident tissue shrinking was observed when ablation was performed during 5–10 min of gastrulation (Figure 5B; Supplementary Movie S2). To further quantify the tissue response to laser ablation, we examined the shape of the treated region over time by tracking the surrounding intact cells before and after laser ablation (Figure 5C; Methods). For each embryo, a region close to the treated region was also analyzed as an internal control (Figure 5C). The change in the width (the size along the medial-lateral axis) of the laser-treated area was used as a readout to determine whether the region underwent shrinking or expansion. The quantification further confirmed the stage specific tissue response to laser ablation. When we ablated embryos at the end of cellularization (immediately before the onset of apical constriction), no obvious tissue shrinking was observed (Supplementary Figure S2A). The tissue width change in the control region and the laser-ablated region was largely comparable. Ablation of embryos during the first 5 min of apical constriction resulted in a mild tissue width reduction over the course of 1 min (Supplementary Figure S2B). In these cases, the steepest rate of tissue width reduction did not occur immediately after laser ablation (Supplementary Figure S2B, orange boxes). This was distinct from the typical exponential decay of tissue recoil upon laser ablation in tensile tissues (Fernandez-Gonzalez et al., 2009; Jodoin et al., 2015; Rauzi et al., 2008). It is therefore unlikely that the mild tissue shrinking observed when ablation was performed within the first 5 min of gastrulation reflects passive tissue material properties. Instead, we speculate that this tissue response was caused by an unknown mechanism that is only active after the onset of gastrulation. When ablation was performed during 5–10 min of gastrulation, the extent of tissue width reduction was substantially increased (Figure 5D). In addition, the tissue response showed a distinct temporal pattern. First, the initial rate of tissue recoil was significantly higher compared to embryos ablated during 0–5 min of gastrulation (Figure 5D). Second, the recoil of the laser-treated area showed an exponential decay over time, with a decay time constant of 27 ± 11 s (mean ± standard deviation, *n* = 7 embryos, Supplementary Figures S2C–E). For comparison, the tissue recoil after laser ablation of tensile tissues in gastrulating *Drosophila* embryos typically decays in the timescale of 10 to 10s of seconds as reported in the literature (Rauzi et al., 2008; Fernandez-Gonzalez et al., 2009; Jodoin et al., 2015). These comparisons suggest that the observed tissue shrinking in embryos during 5–10 min of gastrulation, in particular the initial phase of tissue shrinking (0–15 s), reflects the release of compressive stresses in the tissue upon laser ablation.

The response of the laser ablated area became more complex over longer timescales (40–70 s), which may reflect the normal tissue behavior during the corresponding stage of development (Supplementary Figures S2C,D, cyan boxes) and/or the active tissue response to the laser treatment. To further analyze the tissue response attributed to tissue mechanical properties, we focused our analysis on the initial rate of tissue recoil after laser ablation. The rate of initial tissue response was reported as the rate of change in the width (the size along the medial-lateral axis) of the selected regions within the first 15 s after laser ablation (Figure 5D, green box). The pre-ablation rate was calculated from a 10-s window immediately before the treatment, which revealed no difference between the control and treated regions (Figure 5E). Ablation prior to gastrulation or within the first 5 min of gastrulation resulted in no obvious initial tissue shrinkage. In contrast, the treated region immediately shrunk when ablation was performed 5–10 min into gastrulation, while the control non-treated region did not shrink (Figures 5B,E–G; Supplementary Movie S2). The lack of initial tissue shrinkage when ablation was performed during 0–5 min of gastrulation suggests that the initial tissue shrinking is unlikely a non-specific tissue response to the laser treatment. On the other hand, we could not formally rule out the possibility that certain active tissue behavior that acts over a longer timescale may “contaminate” the measurement of tissue properties during the first 15 s after ablation. For this reason, we use “phenomenological compression” to describe the initial tissue response after laser ablation in embryos during 5–10 min of gastrulation. The presence of tissue compression needs further confirmation using independent approaches in the future. Nevertheless, the emergence of phenomenological compression in the lateral ectoderm during 5–10 min of gastrulation raises the possibility that in-plane compressive stresses are built up in the lateral ectoderm shortly before T_trans_ and function to facilitate mesoderm invagination by triggering mechanical bistability in the mesoderm (Figure 5H).

### Tissue Flow During Ventral Furrow Formation Displays Abrupt Acceleration After T_trans_

**FIGURE 6:**
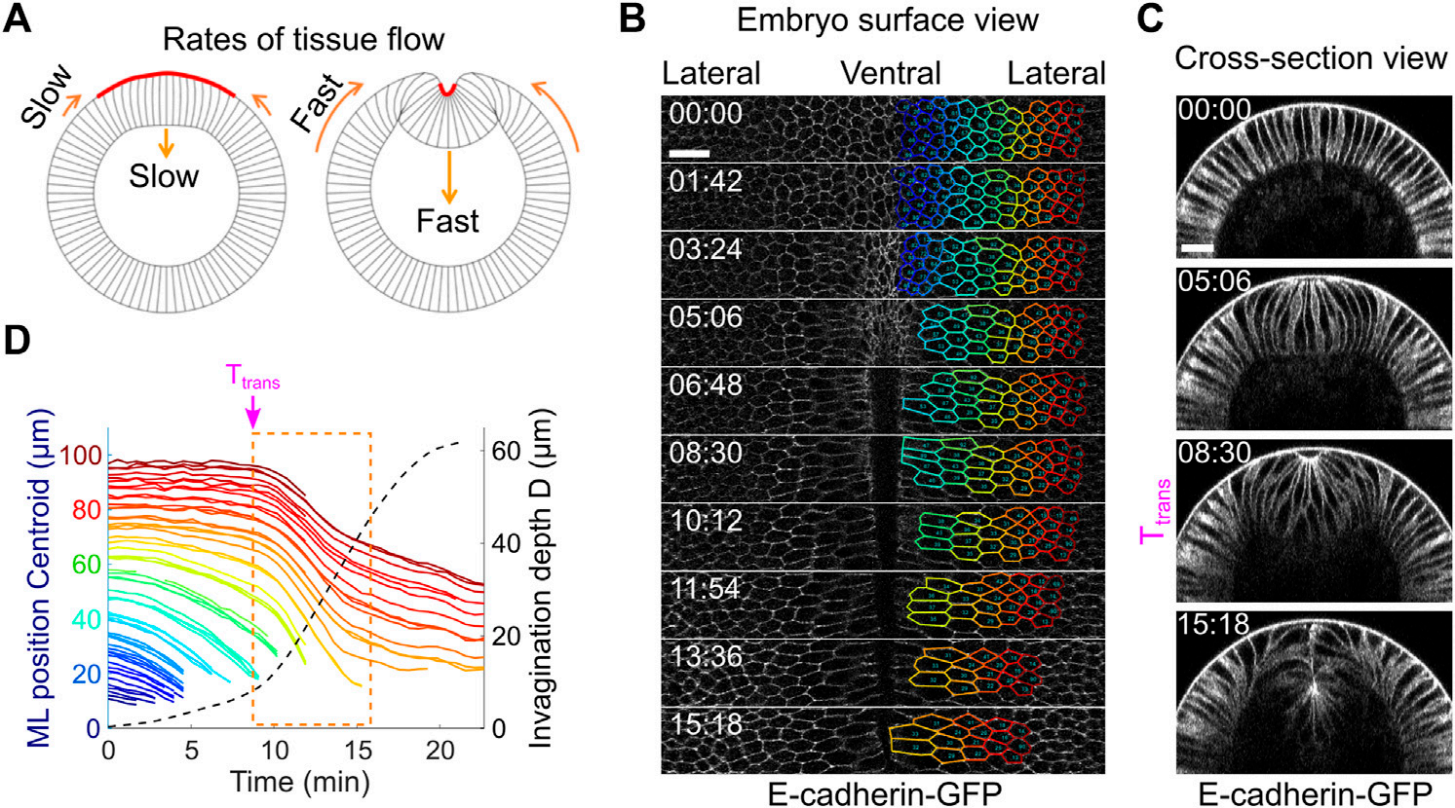
Tissue dynamics during ventral furrow formation. **(A)** Cartoon showing the predicted acceleration of tissue deformation (orange arrows) after T_trans_ that resembles the “snap-through” during an elastic buckling process. **(B)** Surface view of an embryo showing tracked cell outlines during ventral furrow formation. **(C)** Cross-section view of the same embryo shown in **(B)**. **(D)** The relation between cell movement towards ventral midline (solid lines) and the invagination depth D (dotted line) in real embryos. Each solid line represents a single cell, and the color of the lines corresponds to the color of the cell outlines in **(B)**. The rapid acceleration in mesoderm invagination and ectoderm movement happens immediately after T_trans_ (dashed orange box). Shown is a representative measurement out of *N* = 3 embryos. All scale bars: 20 μm.

If *Drosophila* mesoderm invagination is indeed mediated by epithelial buckling, we would expect to observe tissue dynamics that resembles the buckling process. An outstanding signature of elastic buckling is the acceleration of deformation when the system passes through a threshold configuration (“the snap-through”). We therefore measured the speed of tissue movement during ventral furrow formation to test whether such signature exists (Figure 6A). To this end, we segmented ventral and lateral cells at one side of the ventral midline and tracked their motion during gastrulation (Figure 6B; Methods). Our measurements show that there is indeed a rapid acceleration of tissue deformation after the transitional state, as revealed by a steep increase in the rates of both furrow invagination and the ventral movement of the lateral ectoderm (Figures 6C,D, orange box; Supplementary Movie S3). Notably, the onset of the two tissue acceleration events occurred at the same time, suggesting an intimate coupling between the two processes. The observed rapid acceleration in tissue movement is consistent with previous reports (Conte et al., 2012; Polyakov et al., 2014; Rauzi et al., 2015) and is remarkably similar to the buckling transition in compressed elastic sheets.

### Vertex Dynamics Model Incorporating Local Apical Constriction and Global Compression Recapitulates the Buckling-like Tissue Dynamics During Ventral Furrow Formation

**FIGURE 7:**
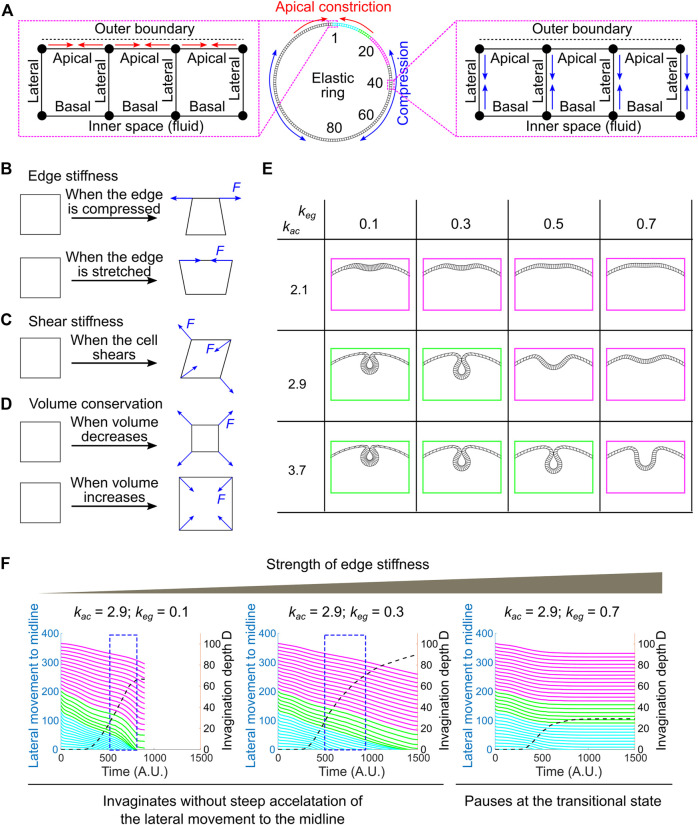
Developing a vertex dynamics model to investigate folding dynamics in a ring-shaped elastic monolayer. **(A)** Vertex dynamics model simulating the behavior of a ring-shaped elastic monolayer undergoing localized apical constriction in the presence or absence of in-plane compressive stresses. The monolayer is discretized into 160 equal units. The IDs of selected units at the right half of the ring are shown. ID 1 marks the midline of the constricting domain. Colored regions are analyzed for their movement during the folding process (panel **F**, similar to the ventral-lateral region analyzed in Figure 6D). Enlarged views: Left, contractions of the apical edges (red arrows) within a designated region of the monolayer results in local apical constriction. Right, shrinking of the lateral edges (blue arrows) outside of the apical constricting domain results in the generation of in-plane compression due to volume conservation. **(B**–**D)** Mechanisms that define the passive mechanical properties of the model. “*F*” denotes restoration forces. **(B)** Edge stiffness. The apical, lateral and basal edges resist stretching or compression like springs. **(C)** Shear stiffness. Shear deformation is dampened through the application of shear restoration forces to resist angle changes between intersected edges. **(D)** Volume conservation. Every unit in the model has a propensity to maintain its volume (cross-section area in 2D) through the implementation of volume restoration forces. **(E)** Under certain combinations of apical constriction $\left(k_{ac}\right)$ and edge stiffness $\left(k_{eg}\right)$ , local apical constriction can trigger invagination of the monolayer in the absence of global compression (*k*_*lc*_ = 0). Only the top region of the model is shown. **(F)** Relationship between invagination depth D (dashed line) and the movement of the lateral non-constricting region (“lateral movement,” solid lines) towards the midline. In the absence of global compression, invagination of the constricting domain (blue box) is no longer associated with a steep acceleration in the lateral movement, which is in contrast to the tight coupling of the two processes in real embryos (Figure 6D). The solid lines are color coded according to the color regime shown in **(A)**.

Finally, we sought to determine whether the buckling-like tissue dynamics observed during ventral furrow formation can arise from a combined action of apical constriction and in-plane compression. To this end, we performed a proof-of-principle analysis using a 2D vertex dynamics model that simulates the behavior of a ring-shaped elastic monolayer confined in a circular shell (Figure 7A). The embryonic epithelium in early embryos is relatively thick (∼32 μm, ∼ 1/3 of the radius of the embryo cross-section). Previous studies suggest that the elastic characteristics of the embryonic epithelium in early *Drosophila* embryos are mainly attributed to the apical cortex (He et al., 2014; Doubrovinski et al., 2017). We therefore simulated a thin sheet (∼4 μm thick) that resembles the apical portion of the epithelium. Since the model did not recapitulate the geometrical characteristics of the actual embryonic epithelium, we were not able to perform a quantitative comparison between the predicted and the observed tissue morphology. However, this simplified approach substantially reduced the complexity of the model, which allowed us to test the central concept of our hypothesis with relatively low computational costs.

In this 2D vertex model, the ring-shaped elastic monolayer is discretized into 160 equal sized units. Each unit has a nearly square shape with two apical nodes and two basal nodes (“vertices”), which define an outer (“apical”) edge, an inner (“basal”) edge and two lateral edges that are shared between the neighboring units (Figure 7A; Supplementary Figure S3; Methods). The apical, lateral and basal edges are elastic and can resist stretching and compression like springs (Figure 7B). The stiffness of the edges is controlled by the spring constant, *k*_*eg*_. In addition, a shear stiffness is implemented to dampen shear deformation of the unit (Figure 7C; *see* justification in Methods and Supplementary Figure S4). Finally, each unit has a propensity to conserve its volume during shape changes, resembling cell volume conservation observed in real embryos (Figure 7D) (Gelbart et al., 2012; Polyakov et al., 2014). Note that our model is elastic instead of viscoelastic. The elasticity assumption has been widely used in the previously published modeling work to simulate ventral furrow formation (Muñoz et al., 2007; Conte et al., 2009; Allena et al., 2010; Hočevar Brezavšček et al., 2012; Polyakov et al., 2014; Rauzi et al., 2015; Heer et al., 2017; Gracia et al., 2019). While this assumption is a simplification of the actual viscoelastic properties of the embryonic tissue, previous rheological studies suggest that elasticity is a reasonable approximation at the time scale relevant to ventral furrow formation (Doubrovinski et al., 2017).

The system is driven out of equilibrium as the apical edges within a designated region of the monolayer (“constricting domain”) undergo contractions. Apical constriction is implemented by adding springs between neighboring apical nodes that have a propensity to shrink (Figure 7A; Methods). The spring constant, *k*_*ac*_, determines the magnitude of apical constriction forces. The spatial and temporal profiles of *k*_*ac*_ are set to resemble the pattern of apical myosin accumulation in real embryos (Supplementary Figures S3A,C; Methods). In order to apply in-plane compressive stresses that resemble the effect of ectodermal compression, we implemented a second type of active forces in the model that drive apical-basal shortening of the lateral edges located outside of the constricting domain (“non-constricting domain”; Figure 7A; Supplementary Figures S3B,D; Methods). Due to volume conservation, apical-basal shortening of the lateral edges will lead to expansion and compression in the planar direction. At each time point, the forces exerted on each vertex from both the active and passive mechanisms are integrated to drive the stepwise movement of the vertex. The movement of all vertices collectively mediates the time evolution of the model.

We first tested whether local apical constriction is sufficient to trigger folding in the elastic monolayer in the absence of ectodermal compression. We found that the behavior of the monolayer is sensitive to the relative strength of the active apical constriction forces (*k*_*ac*_) and the passive stiffness of the edges (*k*_*eg*_). In the regime where *k*_*ac*_ is relatively low and *k*_*eg*_ is relatively high (e.g., [*k*_*ac*_, *k*_*eg*_] = [2.1, 0.3], [2.1, 0.5] or [2.9, 0.7], Figure 7E–magenta), apical constriction caused the formation of a shallow indentation at the site of constriction that resembles the transitional state, but the folding process stopped at the shallow indentation stage without invagination (Figure 7E). In contrast, in the regime where *k*_*ac*_ is relatively high and *k*_*eg*_ is relatively low (e.g., [*k*_*ac*_, *k*_*eg*_] = [2.9, 0.3] and [3.7, 0.5], Figure 7E–green), the model can proceed through the transitional state, allowing the shallow indentation to deepen and eventually form a closed furrow (Figure 7E). However, under these circumstances, the dynamics of the folding process is notably different from that in real embryos. Most prominently, the accelerated invagination (blue boxes in Figure 7F) is no longer associated with a steep acceleration of the movement of the surrounding non-constricting units (Figure 7F). This is in contrast to the tight coupling of the acceleration of the constricting and non-constricting cells in real embryos (Figure 6D). Thus, in the absence of global compression, although apical constriction can mediate the formation of closed furrow under certain [*k*_*ac*_, *k*_*eg*_] combinations, the dynamics of the folding process does not recapitulate the observed tissue dynamics during ventral furrow formation.

**FIGURE 8:**
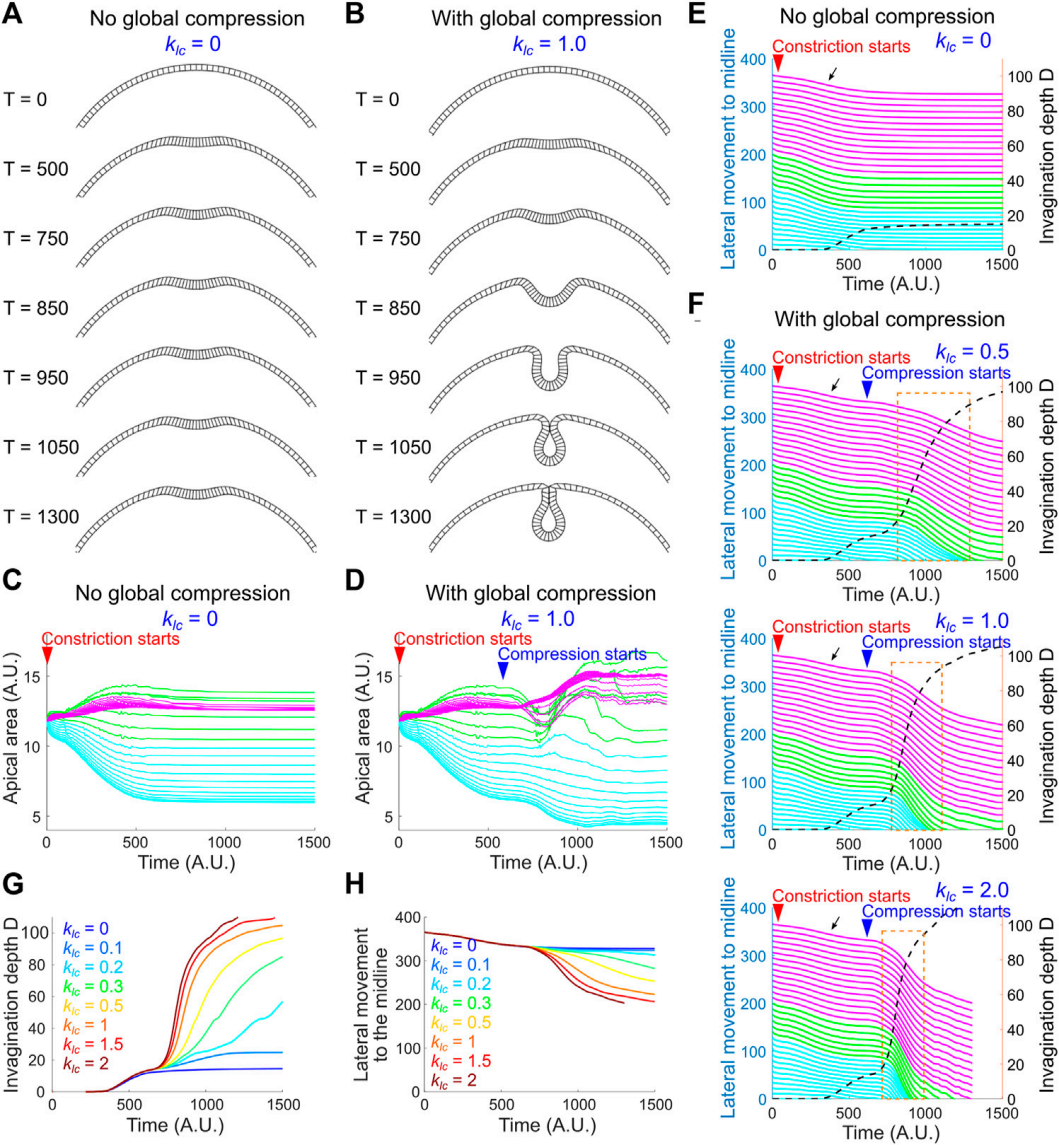
A joint action of local apical constriction and global compression recapitulates real tissue dynamics during ventral furrow formation. **(A**,**B)** Simulated morphological change over time induced by apical constriction with or without global compression. *k*_*ac*_ = 2.1 and *k*_*eg*_ = 0.3 for all simulations shown in this figure. **(C**,**D)** Apical area change with or without global compression. The solid lines in **(C**–**F)** are color coded according to the color regime shown in Figure 7A. **(E**,**F)** Relationship between invagination depth D (dashed line) and the movement of the lateral non-constricting region (“lateral movement,” solid lines) towards the midline in the presence or absence of compressive stresses. Orange box highlights the tight coupling between invagination and the lateral movement during the buckling transition. Black arrow indicates a slight lateral movement during the apical constriction phase which is not observed in real embryos (Figure 6D). Larger $k_{lc}$ (stronger global compression) results in faster invagination and lateral movement during the buckling transition (orange boxes). **(G**,**H)** Simulated invagination depth D and the movement of a laterally localized unit towards the midline under different $k_{lc}$ .

The pause at the small indentation stage in the regime where *k*_*ac*_ is relatively low and *k*_*eg*_ is relatively high resembles the phenotype when the lateral ectodermal cells are disrupted (Figure 2). We therefore asked whether addition of global compression could allow the model to overcome the hindrance at the transitional state. We tested this under the setting of [*k*_*ac*_, *k*_*eg*_] = [2.1, 0.3]. To reflect the observation that the emergence of ectodermal compression is slightly later than the onset of apical constriction (Figure 5), apical-basal shortening of the non-constricting domain was set to occur when the model reaches the transitional state (Methods; Supplementary Figure S3D). Indeed, we found that the emergence of global compression promoted the model to transition from the small indentation state to the fully invaginated state (Figures 8A–D). Notably, this transition is characterized by a steep increase in invagination depth D and a concomitant acceleration of the movement of the lateral non-constricting units (Figures 8E,F). The strong coupling of the movements in the constricting and non-constricting regions closely resembles the actual tissue dynamics during ventral furrow formation. This buckling-like transition is sensitive to the strength of apical-basal shrinking in the non-constricting domain (*k*_*lc*_) that generates in-plane compression (Figures 8F–H). In particular, when *k*_*lc*_ = 0 (no global compression), apical constriction brings the monolayer to the transitional state but is not sufficient to mediate subsequent invagination (Figures 8A,C,E). On the other hand, in the absence of adequate level of apical constriction, compression alone is not sufficient to trigger invagination (data not shown). Thus, our model recapitulates the characteristic tissue dynamics during ventral furrow formation only when the folding of the simulated monolayer is jointly mediated by local apical constriction and global compression. These results demonstrate that the buckling-like tissue dynamics can in theory arise from a combined action of apical constriction and in-plane compression. Taken together, our experimental and modeling analyses support the hypothesis that compression from the ectoderm functions in concert with apical constriction to trigger buckling in the mesoderm during gastrulation.

## Discussion

**FIGURE 9:**
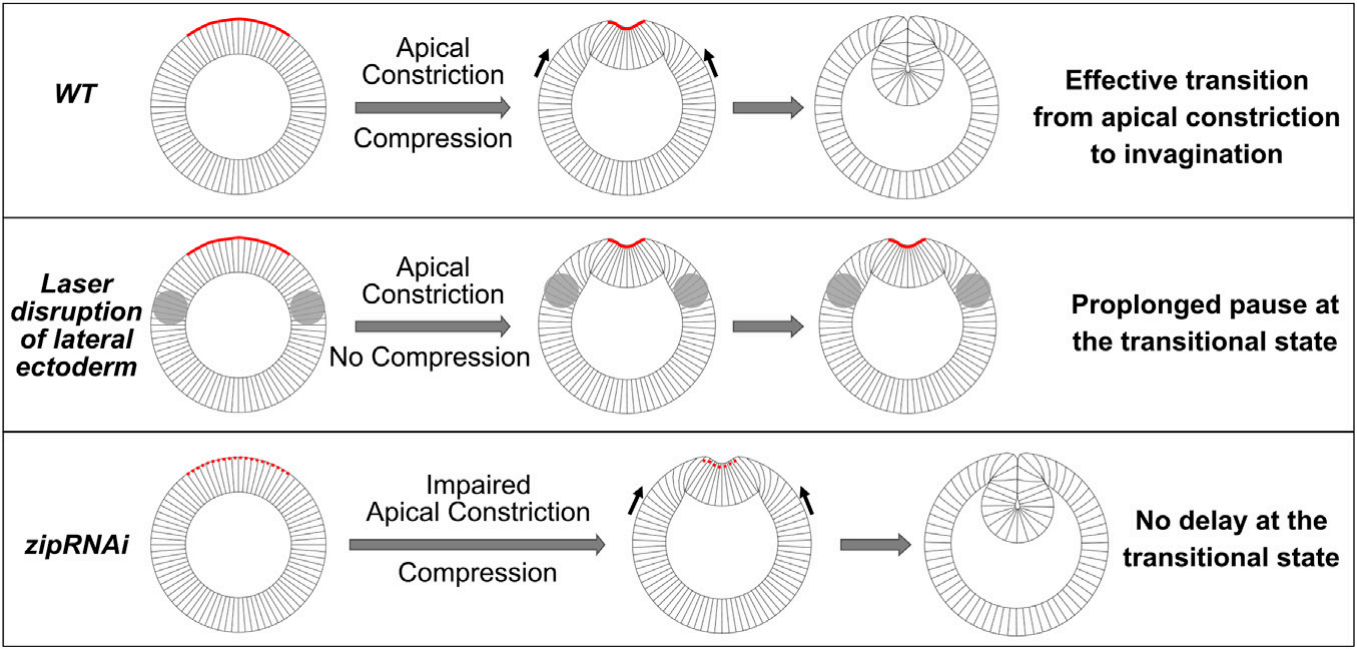
*Drosophila* mesoderm invagination requires a joint action of apical constriction and in-plane compression. Cartoon demonstrating the proposed coordination between apical constriction in the mesoderm and in-plane compression from the ectoderm during mesoderm invagination. We propose that the combined action of apical constriction and compression facilitates mesoderm invagination by triggering epithelial buckling. When ectodermal compression is absent (e.g., when the lateral ectoderm is disrupted), transition from apical constriction to invagination is greatly delayed, and the embryo pauses at the transitional state for an extended time. On the other hand, reducing the rate of apical constriction (e.g., *zip RNAi* embryos) *per se* does not cause delay at the transitional state. In such scenarios, the presence of ectodermal compression facilitates the transition between apical constriction and invagination once the mesoderm epithelium arrives a stereotyped intermediate configuration.

The canonical model for apical constriction-mediated cell sheet folding emphasizes the role of active cell deformation in the constriction domain. In such a view, the transformation of the cells from columnar shape to wedge shape through apical constriction “is sufficient to pull the outer surface of the cell sheet inward and to maintain invagination” (Wolpert et al., 2015). Our recent work on *Drosophila* ventral furrow formation, however, indicates that although actomyosin-mediated active cell deformation is critical during the early, “priming” stage of mesoderm invagination, the subsequent folding step can take place in the absence of actomyosin contractility (Guo et al., 2022). The bipartite tissue response to actomyosin inhibition suggests that the mesoderm epithelium is mechanically bistable during gastrulation. It further leads to the hypothesis that mesoderm invagination is achieved through a buckling process jointly mediated by apical constriction in the mesoderm and compression from the ectoderm (Figure 9). In this work, we developed experimental and theoretical approaches to test this hypothesis. First, through laser-mediated disruption of cell formation in the lateral ectoderm, we show that the presence of the ectodermal tissues surrounding the mesoderm is important for an effective transition from apical constriction to invagination. Second, by developing a laser ablation-dependent approach to detect tissue compression in live embryos, we show that prior to the rapid folding phase of ventral furrow formation, laser ablation in the lateral ectoderm results in tissue response that is anticipated if the tissue is under compression, an observation we describe as “phenomenological compression.” Finally, through quantitative measurements of tissue movement during ventral furrow formation, we observed a steep acceleration of tissue flow immediately after the transitional state. This acceleration of deformation is a hallmark of elastic buckling processes. By developing a 2D vertex dynamics model, we further show that the observed tissue dynamics can be recapitulated by a combined action of local apical constriction and global compression in a simple elastic monolayer. Based on these results, we propose that *Drosophila* mesoderm invagination requires an intimate coupling between the tissue autonomous (mesoderm) and nonautonomous (ectoderm) mechanisms. In the proposed mechanism, the generation of compressive stresses in the plane of epithelial sheet poises the sheet for invagination, whereas the active contractions of the apical and lateral actomyosin cortices within the ventral mesodermal region provide the energy input necessary for the system to transition from the initial state into an intermediate, transitional state, thereby triggering buckling of the epithelium.

A combined action of active and passive mechanisms has also been implicated in other tissue folding processes. For example, during terminal bifurcation of mammalian lung, the developing airway epithelium accumulates compressive stresses due to cell proliferation and gains an intrinsic tendency to buckle (Varner et al., 2015). The bending propensity of the airway epithelium is exploited by the smooth muscle cells located around the incipient bifurcation site, the contraction of which results the inward bending of the tissue between the two prospective lung buds, thereby forming a cleft (Kim et al., 2015). Analogously, we propose that during ventral furrow formation, the buckling propensity of the embryonic epithelium is coupled to the active cell shape change induced by apical constriction, which directs the epithelium to fold specifically at the ventral most region of the embryo. The coupling of active and passive mechanisms may represent a general strategy for cell sheet folding.

While laser ablation has been widely used to detect tissue tension in various contexts, the use of laser ablation to detect tissue compression comes with the caveat that some potential active tissue response to laser treatment or normal morphogenetic tissue movement may “contaminate” the measurement of tissue properties. These active tissue processes may account for the observed mild tissue width reduction after laser ablation during 0–5 min of gastrulation and the more complex tissue response several 10s of seconds after the laser treatment. While it is conceivable that tissue response to laser ablation due to passive mechanical properties should act at a shorter timescale compared to the potential active tissue processes, it is technically challenging to fully tease apart the two types of tissue behaviors from the measured tissue response curve. An important future task is to validate the observed “phenomenological compression” using independent approaches, ideally those that do not cause laser damage in the tissues. Two non-laser-based approaches have been previously used to measure tissue compression *in vivo*. In the first approach, oil microdroplets with defined mechanical properties and coated with adhesion receptor ligands are injected into the tissue of interest. Upon incorporation of the microdroplets into the space between neighboring cells, local anisotropic tensile and compressive stresses can be determined based on the deformation of the microdroplets (Campàs et al., 2014). The second method uses a similar strategy, but instead of using oil microdroplet, fluorescent nanoparticle labeled, elastic round microgel (ERMG) is injected into the tissue. Since ERMG is elastic, it allows the measurement of both isotropic and anisotropic stresses in the tissue (Mohagheghian et al., 2018). Similar to the oil microdroplet approach, the stress measurement using ERMG requires that ERGM being incorporated into the space between cells. While these methods have been successfully applied to measure compressive stresses in mouse and zebrafish embryonic tissues, the application of these methods in early *Drosophila* embryos is difficult due to the challenge of injecting particles larger than a few microns without causing severe damage to the embryo (the size ranges for oil microdroplets and ERGM are 3–40 μm and 15–30 μm, respectively). In addition, since the *Drosophila* blastoderm is composed of tightly packed columnar epithelial cells with a planar cell diameter of 5–10 μm, it is technically difficult to incorporate the oil microdroplets or ERMG into the space between cells. Future development of oil microdroplets or ERMG with smaller sizes may allow the use of these methods to measure tissue compression in gastrulating *Drosophila* embryos.

The potential mechanism that generates the observed “phenomenological compression” in the ectoderm during ventral furrow formation remains to be determined. In certain tissues, compressive stresses are generated as the cells proliferate within a confined boundary (Nelson, 2016). However, no cell division occurs in the embryo during ventral furrow formation (Foe, 1989; Edgar et al., 1994). A possible mechanism for generating compressive stresses is cell shape changes in the ectoderm. It has been reported that ectodermal cells undergo apical-basal shortening during gastrulation (Brodland et al., 2010; Guo et al., 2022). In theory, this cell shape change could generate compressive stresses in the planar direction if the cell volume remains conserved. In addition, previous studies showed that the dorsal cells undergo apical expansion during ventral furrow formation, suggesting a potential cell fate specific mechanism for ectodermal compression (Rauzi et al., 2015). Finally, other morphogenetic processes happening during gastrulation, such as posterior midgut invagination and cephalic furrow formation, might also contribute to the generation of tissue compression (Rauzi et al., 2015). It is possible that the observed in-plane compressive stresses are attributed to multiple processes happening simultaneously in gastrulating embryos. An important research avenue in the future is to elucidate the mechanisms that generate tissue compression and investigate whether compression also plays a role in other tissue folding processes triggered by apical constriction.

The use of tissue compressive stresses may represent a common strategy to facilitate gastrulation in *Drosophila* and related insects. In the midge *Chironomus riparius*, the internalization of the mesoderm occurs through uncoordinated cell ingression that results in a smaller number of internalized cells and a shallower ventral furrow (Urbansky et al., 2016). The difference between *Drosophila* and *Chironomus* is due to the lack of two upstream regulators of actomyosin contractility, Fog and T48, in *Chironomus* (Urbansky et al., 2016). Furrow invagination has also been observed in *Drosophila* mutants where apical constriction is partially defective (e.g., *zip RNAi* embryos). By simple analogy, these observations raise the possibility that ectodermal compression-enabled mechanical bistability may also provide a mechanism to facilitate mesoderm internalization in *Chironomus* embryos, in which the mesoderm precursors do not undergo coherent apical constriction. It is attractive to propose that during evolution, the gain of Fog and T48 activity in presumptive mesoderm cells allow *Drosophila* embryos to increase the efficiency of mesoderm internalization by coupling apical constriction with an evolutionarily more ancient, compression-dependent mechanism for mesoderm internalization.

## Materials and Methods

### Fly Stocks and Genetics

Fly lines containing the following fluorescent markers were used: *Sqh-GFP* (Royou et al., 2002), *Sqh-mCherry* (Martin et al., 2009) and *E-cadherin-GFP* (Oda and Tsukita, 2001).

To generate embryos with *zip* knockdown, *UAS-shRNA-zip* (BDSC 37480, y[1] sc[*] v[1] sev[21]; P{y[+t7.7] v[+t1.8] = TRiP.GL00623}attP40) (Larkin et al., 2021) females were crossed to *67; spider–GFP* males to generate *UAS-shRNA-zip/67; spider–GFP/+* females. Embryos derived from such females were used as *zip* knockdown mutants (*zip-RNAi*). “*67*” refers to the *Maternal-Tubulin-Gal4* on chromosome II (Hunter and Wieschaus, 2000).

### Live Imaging

To prepare embryos for live imaging, embryos were manually staged and collected from apple juice plates, and dechorionated with ∼40% bleach (i.e., ∼3% sodium hypochlorite) in a dark room using an upright Nikon stereo microscope. After dechorionation, embryos were rinsed thoroughly with water, and transferred on a 35 mm glass-bottom dish (MatTek Corporation). Distilled water was then added to the dish well to completely cover the embryos. Live imaging was performed in water at room temperature.

To imaging tissue flow during ventral furrow formation in wildtype embryos (Figure 6), embryos expressing E-cadherin-GFP and Sqh-mCherry were imaged on an Olympus FVMPE-RS multiphoton system equipped with a 25×/1.05 numerical aperture water immersion objective lens. A 3× digital zoom was used. E-cadherin-GFP and Sqh-mCherry were excited with 920-nm and 1040-nm lasers, respectively. A region of interest with the size of 512 pixel (medial-lateral) × 100 pixel (anterior-posterior) (171 μm × 33 μm) at the ventral side of the embryo was imaged. For each time point, a 100-μm z-stack with step size of 1 μm was obtained every 34 s with linearly increased laser intensity over Z.

To examine ventral furrow invagination in *zip RNAi* embryos expressing Spider-GFP (Figure 3), deep tissue live imaging was performed with a custom-built two-photon scanning microscope built around an upright Olympus BX51 (Gelbart et al., 2012). Fluorescence emissions were collected both through an Olympus 40× NA0.8 LUMPlanFl/IR water immersion objective lens) and through an NA1.3 oil condenser lens and were detected with high quantum efficiency GaAsP photomultipliers (Hamamatsu). The microscope was operated by the MATLAB software ScanImage modified to control a piezo objective (PI) and to allow laser power to be increased with greater imaging depth. Images were taken with an excitation wavelength of 920-nm under 3× digital zoom. Stacks of 40 images taken at 2-μm steps were recorded every 8 s. Each image is 256 pixels × 128 pixels (∼0.6 μm/pixel), corresponding to a 150 μm (medial–lateral) × 75 μm (anterior–posterior) region. The imaged regions are approximately centered at the ventral midline.

### Detecting Tissue Compression by Laser Ablation

Embryos expressing E-cadherin-GFP were prepared in the same way as in regular live imaging described above. The embryos were mounted on the coverslip with an orientation that the lateral ectoderm at one side of the embryo was facing the objective. Single color imaging was performed using an Olympus FVMPE-RS multiphoton microscope equipped with a 25×/1.05 numerical aperture water-immersion objective and a 920 nm pulsed laser, which was used for both imaging and laser ablation, with different laser powers. A 3× zoom was used. For detecting compressive stresses in the lateral ectoderm during gastrulation, first, a stack of 51 images taken at 2-μm steps were acquired before the treatment to determine the stage of the embryo. These images encompass the middle portion of the embryo between the anterior and posterior poles and are 512 × 128 pixels (170 × 42 μm) in size. Next, a pre-ablation movie was acquired at a single focal plane ∼17 μm deep from the surface of the embryo (approximately at the middle of the cell height) at a frame rate of 0.93 frame per second for 10 frames. Next, to perform laser cutting, a rectangle region of interest (ROI) approximately 100 × 160 pixels (33 × 53 μm) in size covering approximately 5 × 9 cells was subjected to laser scanning with a higher laser power. The scanning was performed continuously across at a series of z positions from 9 to 27 μm deep from the surface with a step size of 2 μm (covering the middle half of the cell height). The ablation process took approximately 3 s. Finally, to record the response of the tissue immediately after laser ablation, a time lapse movie similar to the setting of the pre-ablation movie was acquired for approximately 2 min. A similar approach was used to detect compressive stresses in tissues with active cell divisions or tensile stresses in tissues undergoing apical constriction, with the following differences. For detecting compressive stresses in tissues with active cell divisions, laser ablation was performed by scanning the laser continuously either across a single z-plane in the middle of the cell height or at a series of z positions that are 4–22 μm deep from the surface with a step size of 2 μm. For detecting tensile stresses in tissues undergoing apical constriction, laser ablation was performed at a single apical z-plane. In all cases, the laser intensity was finetuned such that the laser did not generate obvious damage to the cell membrane, as evidenced by *1*) the lack of burn marks on the tissue or signs of plasma membrane repair and *2*) the readily recovery of the E-cadherin signal on the cell membrane (Supplementary Figure S1). Meanwhile, the laser intensity was high enough to impair the mechanical integrity of the cells, as evidenced by the retraction of surrounding tissues when ablation was performed in regions that are under tension (Figure 4B and data not shown).

### Disrupting Cellularization of Lateral Ectodermal Cells by Laser Ablation

Disruption of the lateral ectodermal cells was carried out on an Olympus FVMPE-RS multiphoton microscope equipped with a 25×/1.05 numerical aperture water-immersion objective and a 920 nm pulsed laser. A 2× digital zoom was used. Embryos at early cellularization stage were mounted on a glass bottom dish with their ventral side facing the objective. First, we focused at a plane in the embryo 60 μm away from the ventral surface. At each lateral side of the embryo, the cellularization front of the ectodermal cells was clearly visible as a line parallel to the surface with a few micrometers’ distance from the surface. Ablation of the cellularization front was performed on both sides of the embryo by scanning a high intensity laser beam across a narrow region below the surface that covers the cellularization front. The same approach was then repeated at a series of z planes from 60 to 110 μm, with a step size of approximately 8–10 μm. After ablation, image stacks (101 z-sections, step size = 1 μm) were acquired under 2× digital zoom every 40 s. The image size was 512 × 128 pixels with a pixel size of 0.5 μm. The total imaged volume is approximately 256 × 64 × 100 μm^3^.

### Image Analysis and Quantification

All image processing and analysis were processed using MATLAB (The MathWorks) and ImageJ (NIH).

To quantify the tissue response after laser ablation (Figure 5), we examined the shape of the laser treated region over time by tracking the surrounding intact cells before and after laser ablation. Cell membrane was segmented based on the E-cadherin-GFP signal using EDGE, a MATLAB-based cell segmentation tool (Gelbart et al., 2012). The two columns of untreated cells immediately next to the medial (proximal to the ventral midline) and lateral (distal to the ventral midline) boundaries of the treated region, respectively, were tracked over time. The change in the average width between these two columns of cells (i.e., the width of the treated region along the medial-lateral axis) over time was used to refer tissue expansion or shrinking after ablation. For each embryo, a non-treated region more anterior or posterior to the ablated region was measured in the same way as a control. The average rate of change in width within 10 s immediately before ablation (“Pre-treatment”) or within 15 s immediately after ablation (“Post-treatment”) was calculated and compared between groups.

To measure the apical constriction rate in the ectoderm disruption experiment (Figure 2), kymographs were generated from the surface views with the cell membrane signal (E-cadherin-GFP) to visualize apical constriction and cell movement over time. An approximately 10-cell wide region that is within the constricting domain and centered at the ventral midline was selected. The width change of the selected region was tracked on the kymograph from the onset of gastrulation to indicate the progress of apical constriction. Similar measurements of apical constriction rate were performed for the *zip RNAi* experiment (Figure 3).

The invagination depth D (i.e., the distance between the vitelline membrane and the apex of the ventral-most cell, Figures 2, 3, 6) was manually measured from the cross-section view of the embryos using ImageJ.

To measure ectodermal cell movement in wildtype embryos during ventral furrow formation (Figures 6B,D), the EDGE program was used to segment the cell outlines from the surface views and to determine the position of the centroid of the segmented cells over time.

### Vertex Dynamics Model on Buckling of an Elastic Monolayer

#### Overview

This 2D vertex dynamics model is designed to investigate the dynamics of an elastic sheet undergoing spatially confined apical constriction in the presence or absence of in-plane compression. The elastic monolayer is composed of 160 equal-size quasi square-shaped units confined in a circular shell, resembling the 2-dimentional cross-section of an elastic tube (Figure 7A). Each unit comprises of an apical edge, a basal edge and two lateral edges connected by nodes (“vertices”). Each lateral edge is shared between two neighboring units. The initial length of the edges is approximately 4% of the radius of the elastic ring (i.e., the monolayer is ∼4 μm thick for the ring that has the same cross-section diameter of a real embryo). The following mechanisms define the “passive” material properties of the monolayer. First, the edges are elastic and can resist stretching and compression like springs (Figure 7B). Second, each unit has shear stiffness that dampens shear deformations (Figure 7C). Finally, the inner space of each unit resists volume changes, resembling the behavior of an incompressible cytoplasm (Figure 7D) (He et al., 2014; Polyakov et al., 2014). The model is driven out of the initial equilibrium configuration by spatially confined apical constriction within a designated region of the monolayer (“constricting domain”). The global in-plane compression is generated through active apical-basal shortening of the lateral edges across the entire non-constricting domain (i.e., regions outside of the constricting domain). Because of the volume conservation propensity, shrinking along the apical-basal axis is expected to generate compression in the planar direction of the monolayer.

#### Passive Components of the Model

The passive components of the model (i.e., the mechanical properties of the monolayer) are determined by the following mechanisms:

##### Edge Stiffness

The apical, basal and lateral edges are elastic and can resist changes in their length. To implement this property in the model, we consider the adjacent nodes as being connected by springs (Figure 7B). When the distance between two adjacent nodes deviates from the resting length of the spring, a restoring force will be applied on the nodes:

$$F_{ij}^{e}=-k_{eg}\left(l_{ij}-l_{0}\right)$$

Here $F_{ij}^{e}$ denotes the elastic force between node i and. $k_{eg}$ is the elastic constant of the edge. $l_{ij}$ and $l_{0}$ represent the current and the equilibrium edge length between node i and j . $l_{0}$ , the equilibrium edge length, equals to the initial edge length. In our model, the spring constant of the apical, lateral and basal edges ( $k_{a}$ , $k_{l}$ and $k_{b}$ , respectively) are set as follows: $\left[k_{a},\;k_{l},\;k_{b}\right]=\left[k_{eg},\;k_{eg},\;\frac{1}{2}k_{eg}\right]$ . Note that $k_{b}$ is set to be half of the value of $k_{a}$ and $k_{l}$ in order to increase the smoothness of the basal surface of the elastic monolayer during simulation. Increasing $k_{b}$ to $k_{eg}$ does not significantly influence the simulation outcome. The impact of varying $k_{eg}$ on the simulation is displayed in Figure 7E.

##### Shear Stiffness

In the actual embryo, shear deformation of cells is not observed in the lateral regions of the embryo during apical constriction (Supplementary Figure S4). In order to constrain the shear deformation in our model, we implemented shear stiffness in all units by creating a “shear resistance force” between each pair of connected edges to resist changes in the angle between them (Figure 7C).

$$F_{i}^{s}=-k_{s}\left(\theta -\pi /2\right)$$

For each pair of edges, the shear resistance force $F_{i}^{s}$ is related to the angle ( θ , in radian) between the two edges connected through the node i . When θ deviates from π/2 , a force will be applied to node i attempting to restore θ to π/2 (Figure 7C). In the model, $k_{s}$ is not treated as a free parameter but is instead set to a fixed value ( $k_{s}$ = 0.5), which is specified to be sufficient to prevent shear deformation in the lateral regions of the model but meanwhile allowing the constricting units to acquire a wedge shape during apical constriction (Supplementary Figure S1).

##### Resistance to Volume Changes

The units in the model have a propensity to maintain their volume (area in the 2D model). To implement this mechanism, when the volume of a unit deviates from the equilibrium level, inward- or outward-pointed forces are applied on all nodes of the unit to restore the volume to the equilibrium level, in a manner analogous to osmotic pressure (Figure 7D). The volume restoration force $F_{i}^{v}$ applied on each node of unit *i* is determined by the difference between the current volume $V_{i}$ and the equilibrium volume $V_{0}$ :

$$F_{i}^{v}=-k_{v}\left(V_{i}-V_{0}\right)$$

$k_{v}$ is the constant for the volume restoration force. In the model, $k_{v}$ is not treated as a free parameter but instead set within a fixed range (0.35–0.45). The values of $k_{v}$ are specified to be high enough to ensure that the volume changes are less than 15% of the equilibrium value. Increasing $k_{v}$ further reduces the magnitude of volume fluctuation away from the equilibrium value, but it also increases computation time while does not significantly alter the simulation outcome.

#### Active Components of the Model

In addition to the “passive” responsive forces mentioned above, we also implemented “active” forces to drive the deformation of the elastic monolayer. These forces are applied by adding additional springs between neighboring nodes.

##### Apical Constriction

The first type of active forces recapitulates apical constriction of the ventral mesodermal cells (Figure 7A). The constriction force is implemented by adding a spring with a shorter equilibrium length between designated apical nodes.

$$F_{ij}^{ac}=-k_{ac}\left(l_{ij}-l_{ac}\right),\;\left(l_{ac}<l_{0}\right)$$

Here, an apical constriction force $F_{ij}^{ac}$ is generated between adjacent apical nodes *i* and *j* when the distance between the two nodes $\left(l_{ij}\right)$ is larger than the equilibrium length of the apical constriction spring $\left(l_{ac}\right)$ , which is set as 1/100 of the initial edge size $l_{0}$ . $k_{ac}$ is the elastic constant of the apical constriction spring. The value of $k_{ac}$ depends on the location of the unit in the elastic ring, with the maximal $k_{ac}$ value $\left(k_{ac}^{max}\right)$ assigned to the apical edges at the midline of the constricting domain, as described below. The impact of varying the magnitude of $k_{ac}$ on the simulation is displayed in Figure 7E.

The spatial distribution of $k_{ac}$ is set to follow a quasi-Gaussian distribution to reflect the graded distribution of apical myosin in real embryos (Supplementary Figure S3A) (Heer et al., 2017; Denk-Lobnig et al., 2021). For a given unit *i* located at the right half of the ring, starting from the midline, $k_{ac}$ is determined by the following function:

$$k_{ac}\left(i\right)=\max\left(C_{ac}e^{\left(-\frac{1}{2\sigma^{2}}{\left(\frac{i-1}{N_{ac}}\right)}^{2}\right)\;}-C_{ac}e^{\left(-\frac{1}{2\sigma^{2}}\left(\frac{N_{ac}-1}{N_{ac}}\right)\right)\;},\;0\right)$$

The term $C_{ac}e^{\left(-\frac{1}{2\sigma^{2}}{\left(\frac{i-1}{N_{ac}}\right)}^{2}\right)\;}$ describes the Gaussian distribution of $k_{ac}$ . $N_{ac}$ is the number of constricting units on each side of the midline, which is set as 16. σ defines the spread of the distribution, which is set as 0.51. The term $C_{ac}e^{\left(-\frac{1}{2\sigma^{2}}\left(\frac{N_{ac}-1}{N_{ac}}\right)\right)\;}$ is a cutoff value which makes $k_{ac}$ outside of the constricting domain zero. The spatial distribution of $k_{ac}$ at the left half of the ring is a mirror image of the right half.

To reflect the gradual accumulation of apical myosin during apical constriction (Martin et al., 2009), the value of $k_{ac}$ is set to increase over time. Specifically, we let $k_{ac}$ to increase from zero to the peak $k_{ac}$ $\left(k_{ac}^{peak}\right)$ between timepoints *t*_*ac1*_ and *t*_*ac2*_, which are set as 0 and 600, respectively (Supplementary Figure S3C). $k_{ac}^{peak}$ is specified according to the position of the cell as described above. For each cell, the temporal profile of $k_{ac}\;$ is defined by the following equation:

$$k_{ac}=\{\begin{array}{cc} 0, & t\leq t_{ac1}\\ \left(9f_{t}^{3}\left(10-15f_{t}+6f_{t}^{2}\right)+1\right)k_{ac}^{peak}, & t_{ac1}<t<t_{ac2}\\ k_{ac}^{peak}, & t\geq t_{ac2} \end{array}$$

Where $f_{t}=\;\frac{t-\;t_{ac1}}{t_{ac2}-t_{ac1}}$

##### Ectodermal Compression

The second type of active forces is the apical basal shortening forces in the non-constricting domain (Figure 7A). In our model, global compression is generated by active shortening of the lateral edges in the non-constricting domain, which results in compression in the planar direction due to the propensity of volume conservation. We implemented this mechanism by adding constricting springs to the lateral sides of the ectodermal cells.

$$F_{ij}^{lc}=-k_{lc}\left(l_{ij}-l_{lc}\right),\;\;\left(l_{lc}<l_{0}\right)\;$$

The shortening force $F_{ij}^{lc}$ will be exerted on nodes *i* and *j* at the two ends of the same lateral edge if the length of this edge $\left(l_{ij}\right)$ is greater than the equilibrium length of the lateral constriction spring $\left(l_{lc}\right)$ , which is set to be 40% of the initial lateral edge size $l_{0}$ . $k_{lc}$ is the elastic constant of the lateral constriction springs. The impact of varying $k_{lc}$ on the outcome of simulation is displayed in Figure 8.

Similar to $k_{ac}$ , the value of $k_{lc}$ also depends on the location of the edge in the elastic ring, such that only the non-constricting domain will undergo active apical-basal shortening (Supplementary Figure S3B). For cells located at the right half of the ring, $k_{lc}$ is defined as follows:

$$k_{lc}\left(i\right)=\{\begin{array}{cc} 0, & \left(1\leq i<16\right)\\ a^{3}\left(10-15a+6a^{2}\right)k_{lc}^{max},\; & \left(16\leq i\leq 24\right)\\ k_{lc}^{max}, & \left(24<i\leq 80\right) \end{array},\mathrm{where}\;a=\left(i-16\right)/8.$$

The spatial distribution of $k_{lc}$ at the left half of the ring is a mirror image of the right half.

To reflect our observation that the onset of ectodermal compression emerges slightly later than the onset of apical constriction (Figure 5), we set the temporal profile of global compression such that it only arises after a certain time. The temporal profile is achieved by allowing $k_{lc}$ to increase from 0 to the peak value between timepoints $t_{lc1}$ and $t_{lc2}$ (Supplementary Figure S3D). $t_{lc1}$ and $t_{lc2}$ are set as 600 and 900, respectively.

$$k_{lc}=\{\begin{array}{cc} 0, & t\leq t_{lc1}\\ \left(f_{t}^{3}\left(10-15f_{t}+6f_{t}^{2}\right)\right)k_{lc}^{peak}, & t_{lc1}<t<t_{lc2}\\ k_{lc}^{peak}, & \;t\geq t_{lc2} \end{array}$$

Where $f_{t}=\;\frac{t-\;t_{lc1}}{t_{lc2}-t_{lc1}}$

#### Geometrical Constraints and Boundary Conditions

##### The Impermeable Edges

We implemented forces analogous to Lennard-Jones forces to prevent the nodes from penetrating any edges. When any given node moves to the close vicinity of an edge that connects two other nodes, a repulsive force is applied to prevent collision of the node with the edge (Supplementary Figure S3E).

$$F_{i}^{LJ}=\frac{k_{LJ}}{r_{i}^{3}}\left(6{\left(\frac{r_{0}}{r_{i}}\right)}^{4}-8{\left(\frac{r_{0}}{r_{i}}\right)}^{2}\right)$$

For any given pair of edge and node *i*, the repulsive force $F^{LJ}$ is related to the shortest distance $\left(r_{i}\right)$ between the node and the edge. $\;k_{LJ}$ is a constant for the repulsive force. $r_{0}$ is a normalizing parameter on distance which is set as two in the simulation. To enhance the stability of the simulation, $F_{i}^{LJ}$ plateaus when $r_{i}$ is below 0.5. Note that $k_{LJ}$ is not treated as a free parameter in the model since the magnitude of this “anti-collision” force does not affect the simulation outcome as far as it falls into the range that is sufficient to prevent node-edge collision.

##### The Impenetrable Outer Shell

The elastic monolayer is confined within a non-penetrable and non-deformable circular boundary that mimics the eggshell of the embryo. When a node moves to the close vicinity of the outer shell, the node will receive a repulsive force that prevent it from getting closer to the shell. The force is similar to $F_{i}^{LJ}$ that prevents nodes from penetrating edges.

##### Inner Hydrostatic Pressure

To mimic the pressure from the yolk region of the embryo, we consider that the space enclosed by the elastic ring is filled with fluid that exerts a hydrostatic pressure on the basal side of the elastic ring. The resulting force exerted on each basal node *i* $\left(F_{i}^{y}\right)$ is normal to the basal surface and is outbound (Supplementary Figure S3F). $F_{i}^{y}$ is set to a fixed value in our simulations (0.25, approximately 1% of the maximal apical constriction force used in Figure 8).

#### Dynamic Components of the Model

At each time point, the forces exerted on each vertex from both the active and passive mechanisms are integrated to drive the stepwise movement of the vertex. The movement of all vertices collectively mediates the time evolution of the model. We consider that the movement occurs in a viscous, low Reynolds number environment where the inertia is negligible (He et al., 2014), and that the forces acting on each node due to the active and passive mechanisms are balanced by viscous drag. The rate at which the node moves is governed by the Stoke’s law, which is proportional to the viscous drag. The simulation ends when a fully closed furrow is formed or when the model reaches a stable intermediate furrow configuration.

### Statistics

Sample sizes for the presented data and methods for statistical comparisons can be found in figure legends. *p* values were calculated using MATLAB ttest2 or ranksum function.

## Data Availability

The raw data supporting the conclusions of this article will be made available by the authors, without undue reservation.
