# Supplementary material for: Evidence for a Role of the Lateral Ectoderm in Drosophila Mesoderm Invagination
Source: Front Cell Dev Biol. 2022 Apr 25;10:867438. doi: 10.3389/fcell.2022.867438 (PMC9081377; doi:10.3389/fcell.2022.867438)
Supplement: Supplementary file 2 [file DataSheet1.pdf]

Evidence for a role of the lateral ectoderm in *Drosophila* mesoderm invagination

Hanqing Guo[1], Shicheng Huang[1] and Bing He[1]*

1. Department of Biological Sciences, Dartmouth College, Hanover, NH

*Correspondence author: Bing He

Email: Bing.He@dartmouth.edu

Supplementary Materials

Supplementary Figures 1 – 4

Supplementary Movies 1 – 3

Supplementary Figure 1

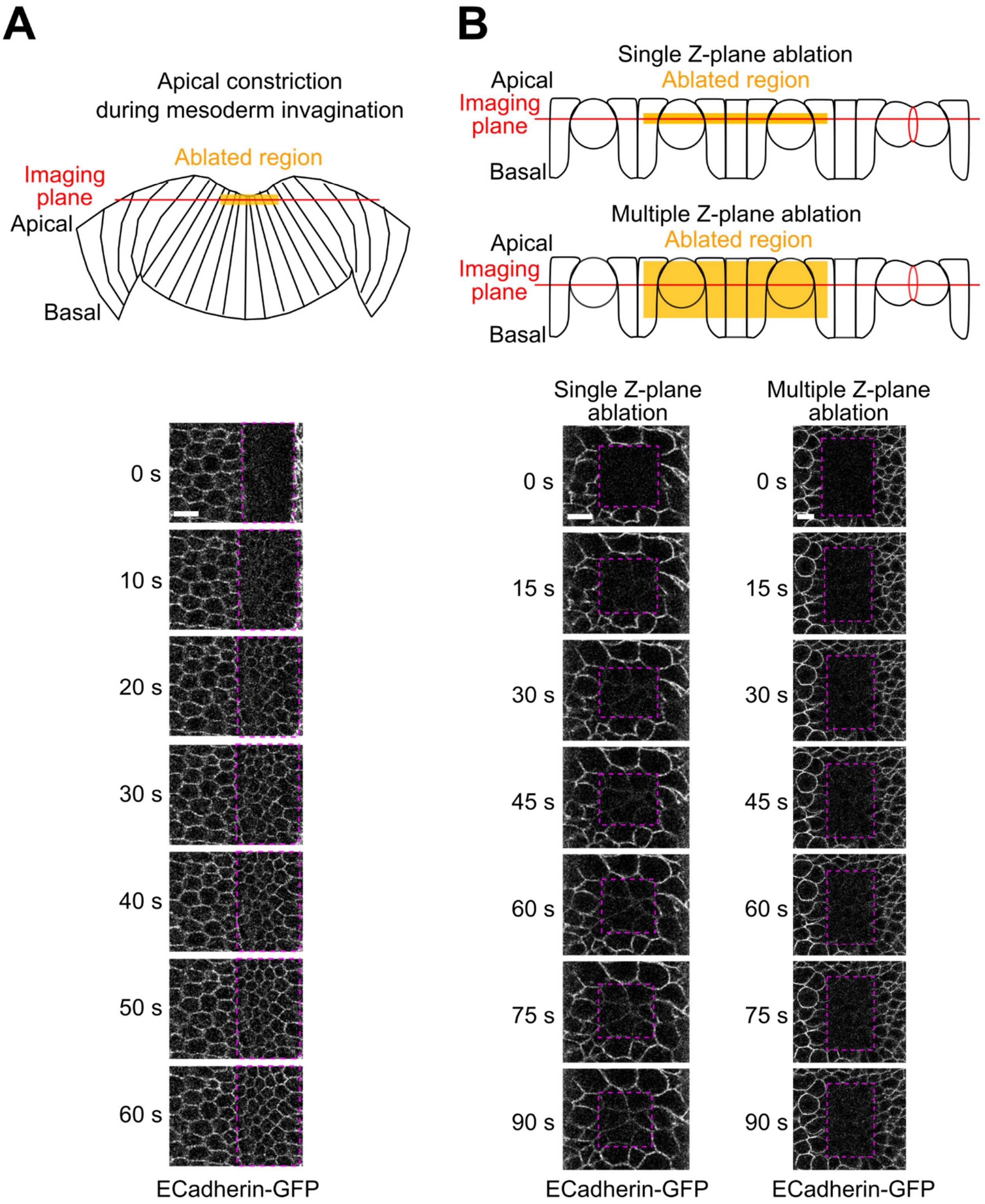

Supplementary Figure 1. Recovery of E-cadherin-GFP signal after laser ablation. (A) Single z-plane laser ablation in the mesodermal tissue during apical constriction. E-cadherin-GFP signal within the laser ablated region (magenta box) quickly recovers after laser ablation. **(B)** Laser ablation within a region of the ectoderm in a stage 8 – 9 embryos where multiple cells are undergoing cell division. Laser ablation is performed either at a single z-plane or at a series of z positions from 4 to 22 μm deep from the surface with a step size of 2 μm. Membrane signal of E-cadherin-GFP quickly recovers after single z-plane laser ablation (left column). In contrast, E-cadherin-GFP signal barely recovers within the same time span after multiple z-plane laser ablation (right column), presumable because a much larger portion of E-cadherin-GFP in the treated cells is bleached by the laser treatment. No burn marks or signs of plasma membrane repair are present in any case described above. All scale bars: 10 μm.

Supplementary Figure 2

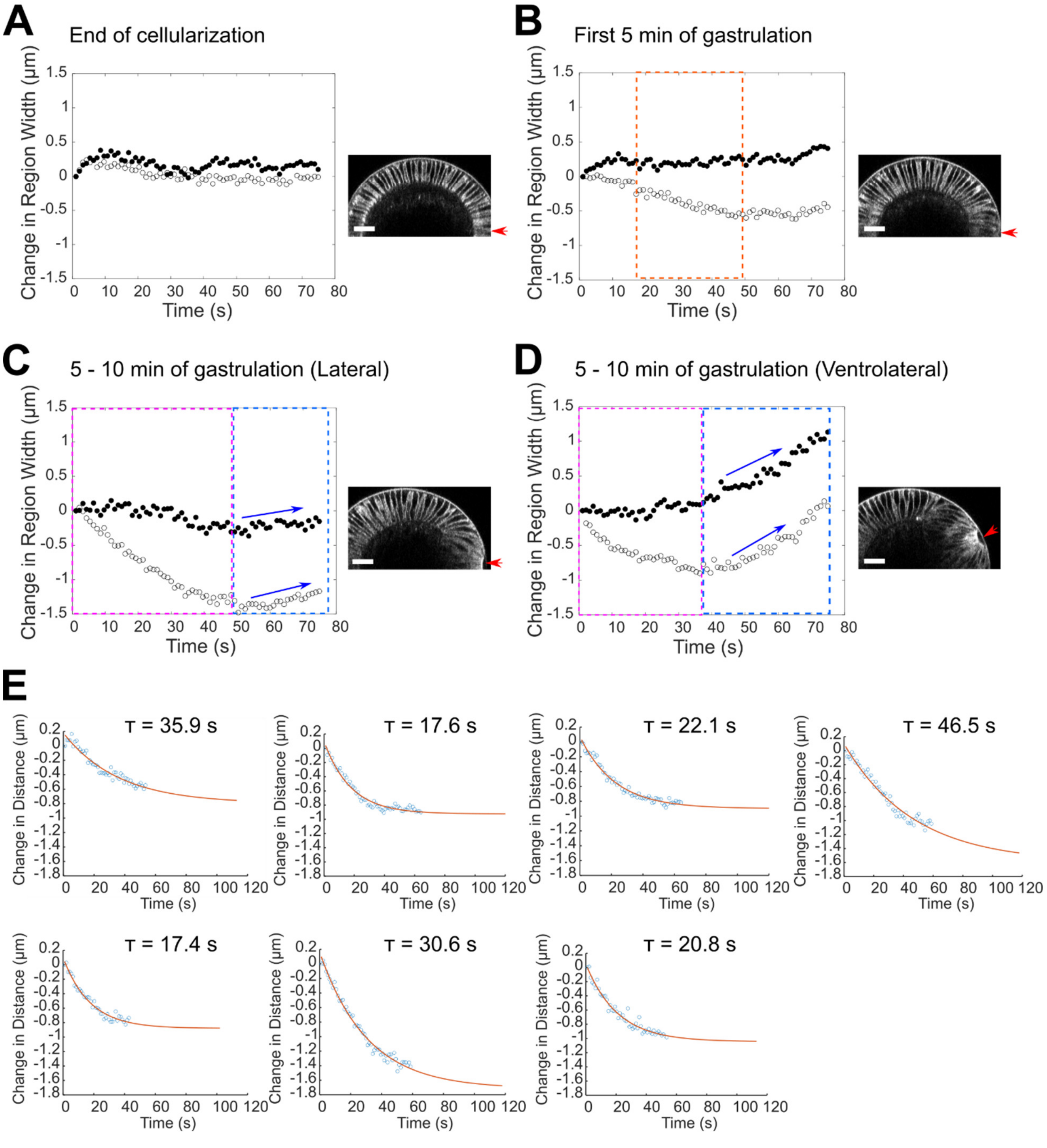

Supplementary Figure 2. Tissue response to laser ablation in embryos ablated at different stages. (A – D) Width change of the control and laser-treated regions over time. (A) A representative embryo ablated near the end of cellularization. No obvious tissue shrinking was observed. The tissue width change in the control region and the laser-ablated region was largely comparable. (B) a representative embryo ablated during 0 – 5 min of gastrulation. Laser ablation resulted in a mild tissue width reduction over the course of one minute. Note that the highest rate of tissue width reduction (orange box) did not occur immediately after laser ablation. (C) a representative embryo ablated during 5 – 10 min of gastrulation. The ectodermal cells located at the lateral side of the embryo was ablated. (D) a representative embryo ablated during 5 – 10 min of gastrulation. The ectodermal cells located at the ventrolateral side of the embryo was ablated. In both (C) and (D), the initial rate of tissue recoil was higher compared to embryos ablated during 0 – 5 min of gastrulation. In addition, the strain of treated area decayed over time (magenta boxes). Note that the response of the laser ablated area became more complex over longer timescales (40 – 70 s), which appears to reflect the normal tissue behavior during the corresponding stage of development (cyan boxes). Blue arrows highlight similar temporal pattern of width change between control and ablated regions after the initial tissue response. The ectodermal cells located at the ventrolateral side of the embryo (D) showed a higher rate of tissue width increase after the initial tissue shrinking compared with the ectodermal cells located at the lateral side of the embryo (C), although in both cases a prominent tissue shrinking was observed immediately after laser ablation. This difference seems to correlate with the distance between the ablated region and the ventral midline (indicated by red arrows), but the actual cause of the difference is unclear. Scale bars: 25 μm. **(E)** Exponential fitting (red curves) of the descending

part of the tissue response curve (blue circles). τ: exponential decay time constant. Only embryos in the category shown in panel c were included in this analysis (n = 7).

Supplemental Figure 3

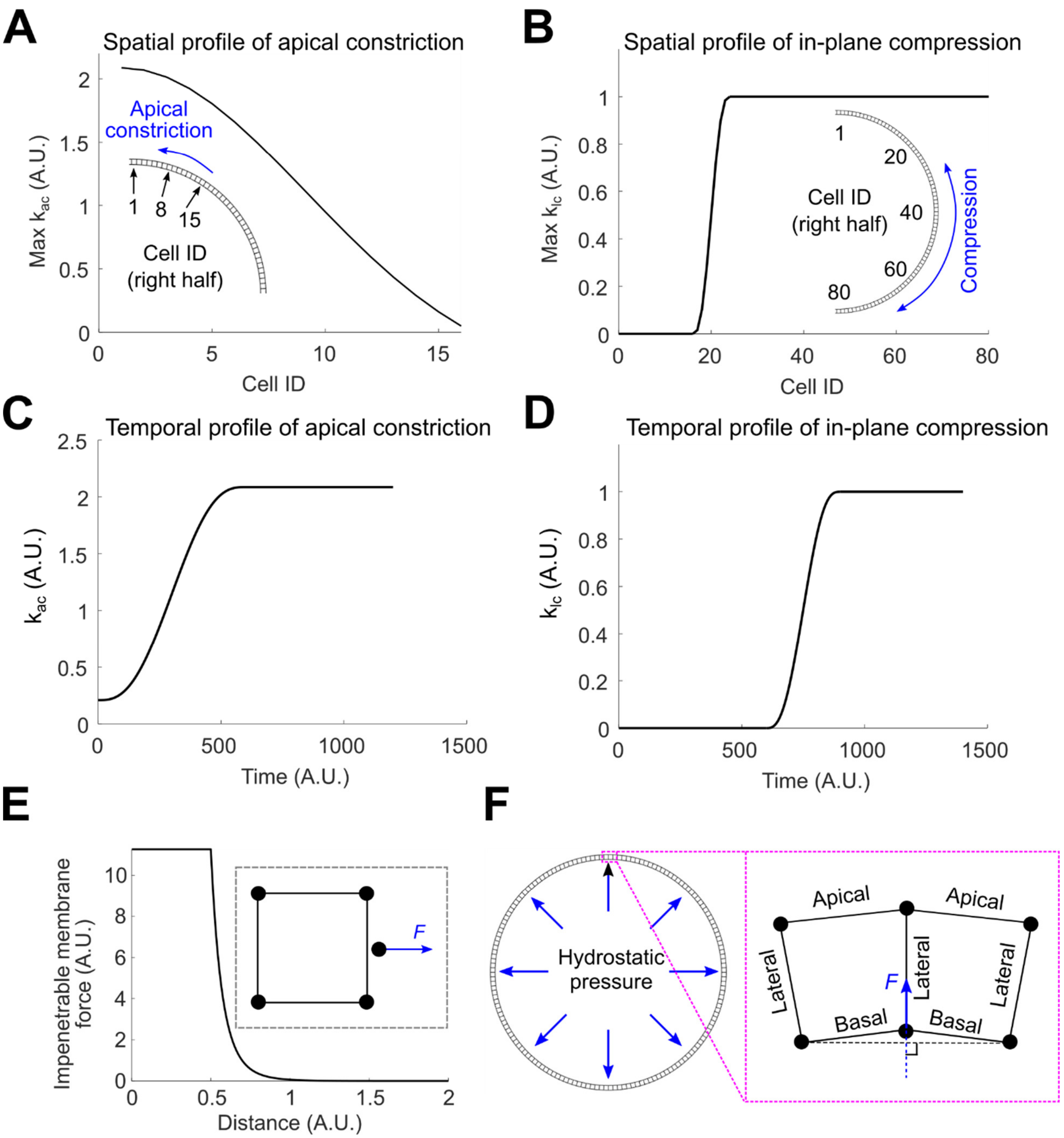

Supplementary Figure 3. Spatial and temporal profiles of the active forces and geometrical constraints. (A) Spatial profile of apical constriction as defined by the quasi-gaussian distribution of maximal k_{ac}. ID 1 refers to the unit at the middle line of the constriction domain. **(B)** Spatial profile of apical-basal shortening defined by k_{lc}. The shortening of lateral edges that generates in-plane compression only occurs in the non-constricting domain. **(C, D)** The temporal profiles of k_{ac} and k_{lc} are set to resemble the observed rise of apical contractility and ectodermal compression during ventral furrow formation. **(E)** Impermeable edges. Repulsive forces ("impermeable membrane forces") are applied to prevent vertices from passing through the outer shell or any edge. When a given vertex becomes very close to the outer shell or an edge that connects two other vertices, a repulsive force analogous to Lennard-Jones force is applied to the vertex to prevent collision. **(F)** Inner pressure. The space enclosed by the elastic monolayer is filled with fluid that exerts hydrostatic pressure on the inner side of the monolayer (left panel). This inner pressure resembles the pressure from the yolk in real embryos. The yolk pressure forces are outbound and are normal to the basal surface (right panel).

Supplemental Figure 4

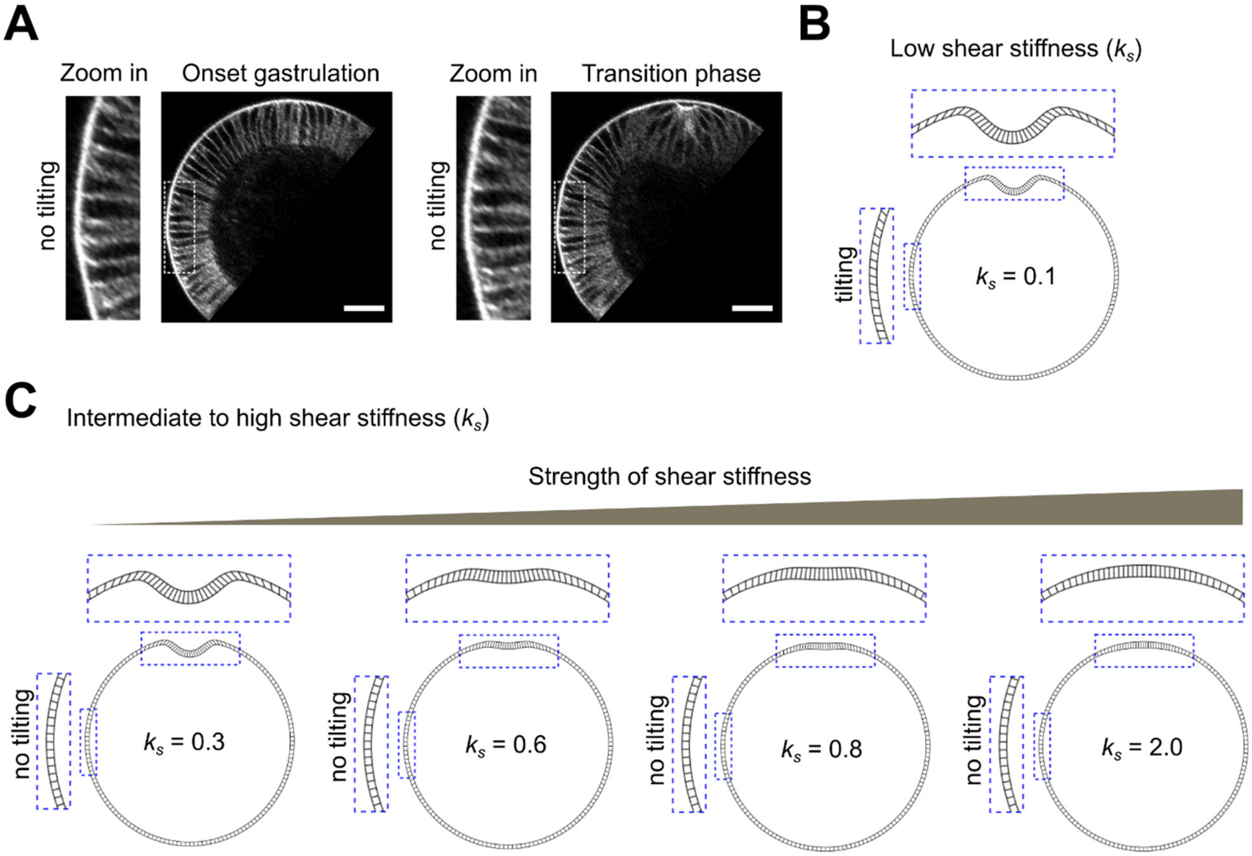

Supplementary Figure 4. The impact of varying shear stiffness on the model. (A) A representative embryo expressing E-cadherin-GFP at the onset (left) and the transition phase (right) of ventral furrow formation. Enlarged view showing the apical portion of the lateral ectoderm. No cell tilting occurs at the lateral side of the embryo during apical constriction. **(B)** At low shear stiffness, the units on the lateral side of the model undergo tilting during apical constriction. k_{ac} = 2.1, k_{eg} = 0.3, and k_{lc} = 0 (same in C)**. (C)** At intermediate to high shear stiffness, no tilting occurs at the lateral side of the model. However, apical constriction is increasingly obstructed as shear stiffness increases. k_s is set at a fixed value of 0.5 for the simulations presented in the main text.

Movie Legends

Movie 1. Detecting tissue compression generated by dividing cells using laser microdissection. Shown are the lateral region of one representative E-cadherin-GFP expressing embryo immediately before ("Pre") and after ("Post") laser microdissection. Laser microdissection applied to a 5×9 cells region for approximately 3 seconds. For both "Pre" and "Post" stages, an overlay between the cell outline at the current time (green) and the first frame (magenta) is shown to highlight the changes in tissue shape after laser microdissection. An immediate shrinkage of the treated region is observed in "Post" stage compared to the relative stable cell position observed in the "Pre" stage. The gradual recovery of the membrane signal of E-cadherin-GFP and the lack of sign of cell membrane repair suggests that the cell membrane is not damaged.

Movie 2. Detecting tissue compression in the lateral ectoderm using laser microdissection. Shown are the lateral region of two E-cadherin-GFP expressing embryos immediately before ("Pre") and after ("Post") laser microdissection applied to a 5×9 cells region for approximately 3 seconds. For the "Post" stage of the movie, an overlay between the cell outline at the current time (green) and that at time zero (magenta) is shown to highlight changes in tissue shape after laser treatment. An immediate shrinkage of the treated region is observed in the embryo 5 – 10 min into gastrulation (right), but not in the embryo less than 5 min into gastrulation (left). The cross-section view of the same embryo immediately before laser dissection is shown at the bottom inset to indicate the stage. The gradual recovery of the membrane signal of E-cadherin-GFP and the lack of sign of membrane repair suggest that the cell membrane is not damaged.

Movie 3. Acceleration of tissue flow during ventral furrow formation. Shown are the ventral surface view and the cross-section view of the same embryo in the process of ventral furrow formation. A selected group of cells are tracked and outlines in the surface view, and their displacement towards the ventral midline over time is plotted along with the time evolution of invagination depth D. The measurements reveal a rapid acceleration of tissue deformation at the lengthening-shortening transition, which is reflected by a steep increase in the rates of furrow invagination and the ventral movement of the lateral ectoderm.
